# Comprehensive analysis of metabolism-related gene biomarkers reveals their impact on the diagnosis and prognosis of triple-negative breast cancer

**DOI:** 10.1186/s12885-025-14053-8

**Published:** 2025-04-11

**Authors:** Weibin Ren, Yuyun Yu, Tao Wang, Xueyao Wang, Kunkai Su, Yanbo Wang, Wenjie Tang, Miaomiao Liu, Yanhui Zhang, Long Yang, Hongyan Diao

**Affiliations:** 1 Jinan Microecological Biomedicine Shandong Laboratory, Jinan, 250117 Shandong China; 2 The First Affiliated Hospital, Zhejiang University School of Medicine, Hangzhou, Zhejiang China

**Keywords:** Triple-negative breast cancer (TNBC), Metabolic signatures, Prognostic, Immune infiltration, Drug sensitivity

## Abstract

**Background:**

Triple-negative breast cancer (TNBC) is an aggressive subtype of breast cancer characterized by poor prognosis and limited treatment options, which underscores the urgency of the discovery of new biomarkers. Metabolic reprogramming is a hallmark of cancer and is expected to serve as a strong predictive biomarker for breast cancer.

**Methods:**

We integrated RNA expression data and clinical information from The Cancer Genome Atlas (TCGA) and Gene Expression Omnibus (GEO) databases to explore the associations between metabolism-related gene expression and TNBC prognosis. Our comprehensive approach included differential expression analysis, enrichment analysis, Cox regression analysis, machine learning, and in vitro experimental validation.

**Results:**

We identified five pivotal genes—SDS, RDH12, IDO1, GLDC, and ALOX12B—that were significantly correlated with the prognosis of TNBC patients. A prognostic model incorporating these genes was developed and validated in an independent patient cohort. The model demonstrated predictive validity, as TNBC patients classified into the high-risk group exhibited significantly poorer prognoses. Additionally, utilizing the risk model, we evaluated the mutational landscape, immune infiltration, immunotherapy response, and drug sensitivity in TNBC, providing insights into potential therapeutic strategies.

**Conclusions:**

This study established a robust prognostic model capable of accurately predicting clinical outcomes and metastasis, which could aid in personalized clinical decision-making.

**Supplementary Information:**

The online version contains supplementary material available at 10.1186/s12885-025-14053-8.

## Background

Triple-negative breast cancer (TNBC) is a subtype of breast cancer with unique biological characteristics that accounts for 10–20% of all breast cancers. It is characterized by a lack of expression of estrogen receptor (ER), progesterone receptor (PR), and human epidermal growth factor receptor 2 (HER2) in tumor cells. Owing to the absence of clear therapeutic targets, TNBC is insensitive to traditional endocrine therapy and HER2-targeted treatments, which limits treatment options and leads to a relatively poor prognosis [1–4]. The biological behavior and clinical presentation of TNBC significantly differ from those of other subtypes of breast cancer, with a higher risk of recurrence and earlier timing of relapse. Additionally, TNBC is more common in younger women and certain racial groups, and immune cell infiltration in the tumor microenvironment also exhibits characteristics different from those of other subtypes [1, 2, 5]. In recent years, with in-depth research into the molecular heterogeneity of TNBC, various molecular subtypes, which significantly differ in tumor aggressiveness, metastatic potential, and treatment response, have been revealed [3, 6–9]. These findings provide new perspectives for precision therapy in TNBC and have fostered the development of novel treatment strategies targeting specific molecular targets. Concurrently, the potential of immunotherapy in TNBC has gradually been recognized, offering new therapeutic hope for these patients. Despite the advent of targeted therapies, including PARP inhibitors that have been approved for BRCA-mutated TNBC [10], and the significant promise shown by immune modulators, these treatment approaches are still in their early stages of development [11]. Currently, chemotherapy remains the standard treatment for TNBC [12]. Therefore, there is an urgent need to identify and validate new prognostic markers to improve treatment options and prognostic assessment for TNBC patients, thereby promoting the development of more precise and effective treatment strategies.

Recent studies have revealed a close link between tumor growth and metabolic pathways, especially in TNBC, where tumor cells exhibit metabolic characteristics different from those of normal cells, including unique patterns of glucose, fatty acid, and amino acid metabolism [13]. This metabolic reprogramming not only is a hallmark of cancer but also leads to complex metabolic interactions between immune cells, cancer stem cells, the tumor microenvironment, and the gut microbiota, which have profound effects on treatment response and clinical outcomes [13–15]. Metabolic heterogeneity is regarded as a promising anticancer strategy, and a deep understanding of the molecular changes caused by metabolic reprogramming is crucial for advancing the development of targeted therapies [16]. In TNBC, the key role of metabolic reprogramming further emphasizes the potential association between the abnormal expression of metabolism-related genes and patient prognosis. However, current research on the role of metabolic genes in TNBC prognosis is relatively limited, which highlights the need for in-depth studies of these genes and the development of prognosis models based on metabolism-related genes.

In this study, we constructed and validated a prediction model based on metabolism-related genes aimed at predicting the clinical prognosis of TNBC patients. By conducting an in-depth analysis of metabolism-related differentially expressed genes (DEGs) in the TCGA-TNBC dataset, we ultimately identified five key genes that have significant predictive value in the prognostic assessment of TNBC. The characteristics of metabolic genes not only reveal the complexity of metabolic heterogeneity in TNBC but also provide possibilities for the development of new therapeutic strategies and personalized treatment plans. Our research emphasizes the role of metabolic reprogramming in the development of TNBC and provides new molecular prognostic markers and potential therapeutic targets for future research and clinical practice. With this approach, we hope to provide more accurate prognostic assessments and more effective treatment options for TNBC patients.

## Methods

### Data collection, preprocessing, and acquisition of metabolism-related genes

The RNA expression data and clinical and follow-up information of 158 patients with TNBC were downloaded from the TCGA cohort (https://portal.gdc.cancer.gov). Given that the original data were log2(x + 1) transformed RSEM normalized counts, the data were converted to Count values (using the formula a = a²-1) for subsequent analysis. Additionally, microarray gene expression data along with clinical and follow-up information for two external validation cohorts were obtained from the Gene Expression Omnibus (GEO) database (https://www.ncbi.nlm.nih.gov/geo/). Specifically, the GSE58812 cohort (GPL570 Affymetrix HG-U133 Plus 2.0 platform) comprised 107 TNBC samples [17], while the GSE21653 dataset (GPL570 Affymetrix HG-U133 Plus 2.0 platform) contained 85 TNBC cases [18, 19]. This study adheres to the data access policies and publication guidelines of the TCGA and GEO.

A total of 1,660 genes involved in 86 metabolic pathways were downloaded from the Kyoto Encyclopedia of Genes and Genomes (KEGG) database (https://www.genome.jp/kegg/) [20], and detailed information and classification of metabolism-related genes can be found in Supplementary Table S1.

### Identification of differentially expressed metabolism-related genes

Differential expression of metabolism-associated genes in 158 TNBC samples and 114 normal samples was identified via the ‘DESeq2’ [21] R package, with thresholds of|log2-fold change (FC)| > 1 and *P* < 0.05 for selection. Volcano plots for metabolism-associated genes were generated via the ‘ggpubr’ R package, and heatmaps for differentially expressed metabolism-associated genes were constructed via the ‘pheatmap’ R package.

### Functional enrichment analysis

Gene Ontology (GO) and Kyoto Encyclopedia of Genes and Genomes (KEGG) enrichment analyses of the DEGs were performed, and the results were visualized via the R package clusterProfiler [22]. Gene set enrichment analysis (GSEA) was conducted to characterize the biological functions of the high- and low-risk groups, utilizing “c2.cp.kegg.v7.5.1.entrez.gmt” as the reference database, with thresholds of|NES| > 1.5 and FDR Q value < 0.05 for selection [23].

### Identification of prognosis-associated key metabolic genes

To elucidate the relationship between the expression levels of metabolic genes and overall survival in TNBC patients, we initially conducted univariate Cox regression analysis on metabolism-related differentially expressed genes (DEGs) within the TCGA-TNBC dataset via the “survival” R package, with a *P* value < 0.05 for significant filtering for further analysis. We subsequently employed the “glmnet” R package with least absolute shrinkage and selection operator (LASSO) Cox regression to mitigate gene collinearity and reduce the number of candidate genes [24]. Ultimately, multivariate Cox regression analysis was performed to identify the final set of candidate genes.

### Construction and validation of a prognostic model based on metabolism-associated genes

The risk score was calculated via the formula Risk score = $\:{\sum\:}_{i}^{n}XiYi$, where *Xi* represents the coefficients of metabolism-associated genes identified via multivariate Cox regression analysis and *Yi* denotes the expression levels of the corresponding genes [25–27]. This computation was based on the normalized mRNA expression data of the TCGA-TNBC dataset. Patients with TNBC were categorized into high- and low-risk groups according to the median risk score, and the overall survival of these groups was analyzed. To evaluate the prognostic performance of the model, receiver operating characteristic (ROC) curves were generated via the “timeROC” R package. The accuracy of the model was validated in a TNBC cohort from the GEO database. Furthermore, to determine whether the risk score is an independent prognostic factor for overall survival in TNBC patients within the TCGA-TNBC dataset, univariate and multivariate Cox regression analyses were conducted, with covariates including age and tumor stage.

### Analysis of mutations in high- and low-risk groups

Employing the “maftools” R package, we performed an analysis of single nucleotide polymorphisms (SNPs) and mutational burden in samples categorized into high- and low-risk groups [28].

### Analysis of immune therapy response and drug sensitivity in samples from high- and low-risk groups

Using the online tool TIDE (http://tide.dfci.harvard.edu/), we calculated the scores for samples in both the high- and low-risk groups. Immunophenotype scores (IPSs) for breast cancer patients were downloaded from The Cancer Immunome Atlas (TCIA, https://tcia.at/home), and IPSs specific to TNBC patients were extracted for the comparison of IPS scores between the high- and low-risk groups. Drug sensitivity for both groups was predicted via the “oncoPredict” R package [29], with the “GDSC2_Expr.rds” and “GDSC2_Res.rds” datasets from the Genomics of Drug Sensitivity in Cancer (GDSC) database serving as the training sets. Violin and box plots were generated via the “ggpubr” package.

### Analysis of the tumor microenvironment and immune infiltration in samples from the high- and low-risk groups

The immune infiltration of tumors within different risk subgroups was assessed via the ESTIMATE algorithm [29], CIBERSORT algorithm [30], and ssGSEA algorithm [31].

### RNA extraction, cDNA synthesis and RT‒qPCR

Total RNA was extracted from the breast cancer cell lines via the TRIzol reagent (Invitrogen, Catalog Number: 15596026CN). The RNA samples were subsequently reverse transcribed into cDNA via the Hifair^®^ II 1st Strand cDNA Synthesis Kit (YEASEN, China, Catalog Number: 11119ES60). RT‒qPCR was carried out with the Hieff^®^ qPCR SYBR Green Master Mix (No Rox) kit (YEASEN, China, Catalog Number: 11204ES08) following the manufacturer’s instructions. After normalization to β-actin, the expression levels of all target genes were calculated via the 2^-ΔΔCt^ method after the reactions were performed in triplicate. The sequences of primers used were as follows: IDO1-F: 5’-GCAAATGCAAGAACGGGACA-3’ and IDO1-R: 5’- ATAGCTGGGGGTTGCCTTTC-3’; SDS-F: 5’- CTGCCCAAGATCACCAGTGT-3’ and SDS-R: 5’- GCCTCCTGGTCCGAGATAAC-3’; GLDC-F: 5’-GTACAGCTCAGGCCCTCTTG-3’ and GLDC-R: TGCTCGCTTGAGACCTTCTG.

### Cell culture and SiRNA transfection

The breast cancer cell lines MDA-MB-231 and MCF-7, as well as the normal human breast epithelial cell line MCF10A, were originally obtained from the American Type Culture Collection (ATCC) and subsequently provided by the First Affiliated Hospital of Zhejiang University. The cells were maintained in DMEM (Gibco, Thermo Fisher Scientific, Catalog Number: AJ30728130) supplemented with 10% fetal bovine serum (FBS) (Gibco, Thermo Fisher Scientific, Catalog Number: E607016-3000) and 1% penicillin/streptomycin (Gibco, Thermo Fisher Scientific, Catalog Number: E607011-0100) at 37°C in a humidified atmosphere containing 5% CO_2_ [32]. The siRNA sequences used to target IDO1 were as follows: sense: 5’-GGACAAUCAGUAAAGAGUA-3’; antisense: 5’-UACUCUUUACUGAUUGUCC-3’. And the siRNA-NC sequences were as follows: sense: 5’-UUCUCCGAACGUGUCACGU-3’; antisense: 5’- ACGUGACACGUUCGGAGAA-3’. The siRNA transfection experiment was performed as follows: First, siRNA was diluted to a working concentration of 30 nM using serum-free medium. The diluted siRNA was then mixed with Transmate transfection reagent (Sangon Biotech (Shanghai) Co., Ltd. Catalog Number: E607405) at a 1:1 ratio, gently mixed, and incubated at room temperature for 10 min to form the siRNA/Transmate complex. The complex was added dropwise to the cell culture medium, and the culture plate was gently shaken to ensure even distribution. The cells were then returned to a 37 °C, 5% CO₂ incubator for continued cultivation. After 6 h of transfection, the medium was replaced with fresh complete medium to remove the transfection complex and reduce cytotoxicity. The cells were further cultured for 24 h, after which samples were collected, and the mRNA levels of the target gene were detected by qPCR to evaluate transfection efficiency.

### Cell counting Kit-8 (CCK-8) assay

The cells were seeded into 96-well plates (1 × 10^3^), and 10 µl of CCK-8 solution (Yeasen Biotechnology Co., Ltd., Shanghai, Catalog Number: 40203ES60) was added at 24, 48, and 72 h posttransfection. After incubation at 37 °C for 2 h, the absorbance at 450 nm was measured via a TECAN Infinite 200 microplate reader [33].

### Transwell assay

The cells (1 × 10^4^) were seeded into migration chambers (Cellprobio Biotechnology (Suzhou) Co., Ltd.), and medium containing 10% fetal bovine serum (FBS) was added as a chemoattractant. After 24 h, methanol was added to fix the cells, which were then stained with 0.1% crystal violet (YEASEN, China, Catalog Number: 60505ES25) and counted [33].

### Wound-healing assay

The cells were seeded into a cell culture plate (Cellprobio Biotechnology (Suzhou) Co., Ltd.) and grown to form a confluent monolayer. A linear wound was generated by scraping the cells with the tip of a 1-mL pipette. After 24 h, the migrating cells were imaged via an inverted microscope [33].

### Statistical analysis

In this study, all the statistical analyses were performed via R software (version 4.3.1) and GraphPad Prism software (version 9.0). Comparisons between two groups were made via t tests, whereas comparisons among multiple groups were conducted via one-way ANOVA. A *P* value < 0.05 was considered to indicate statistical significance.

## Results

### Identification of metabolic genes differentially expressed in TNBC patients

**Fig. 1:**
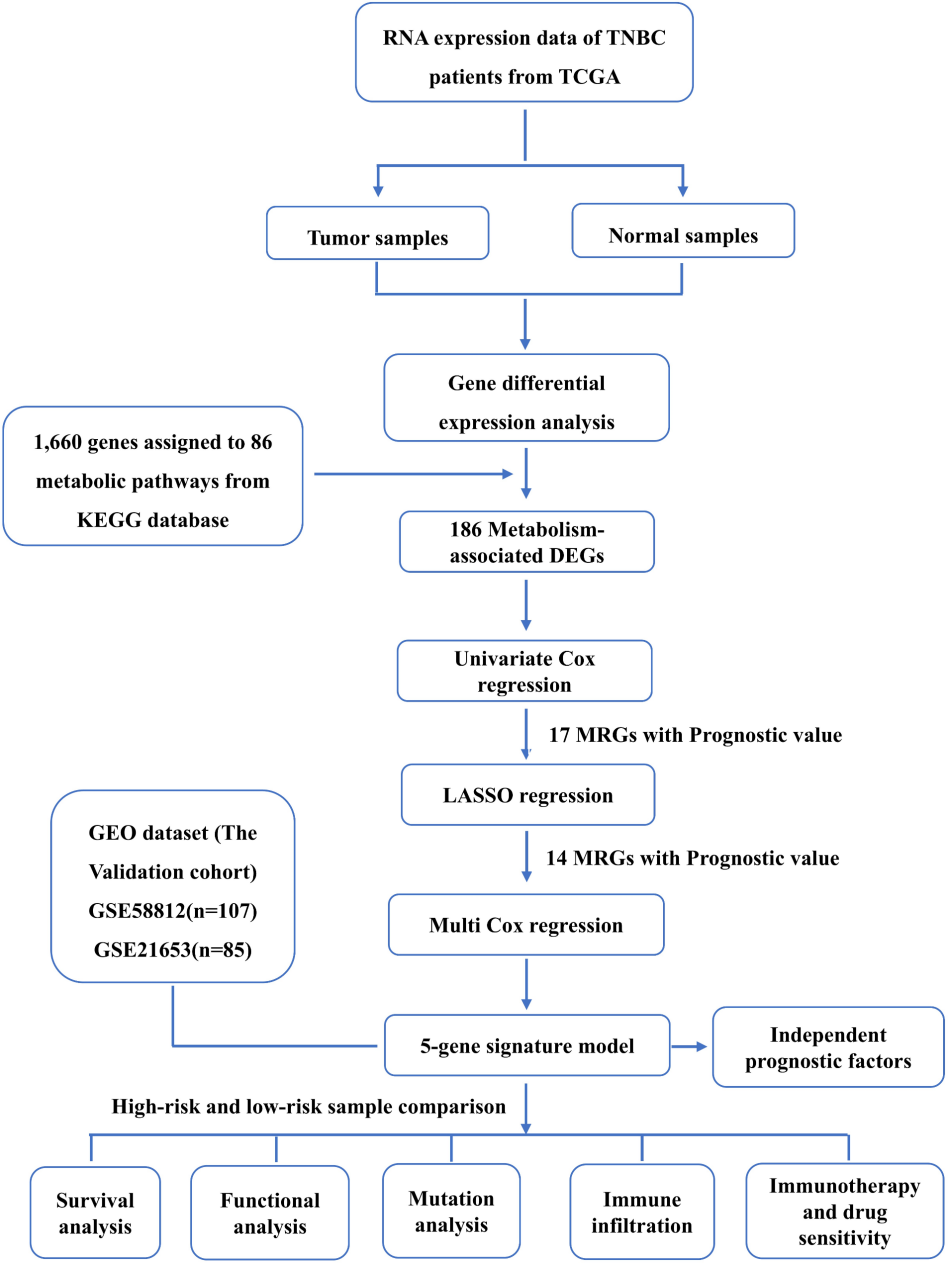
Research flowchart of this study

**Fig. 2:**
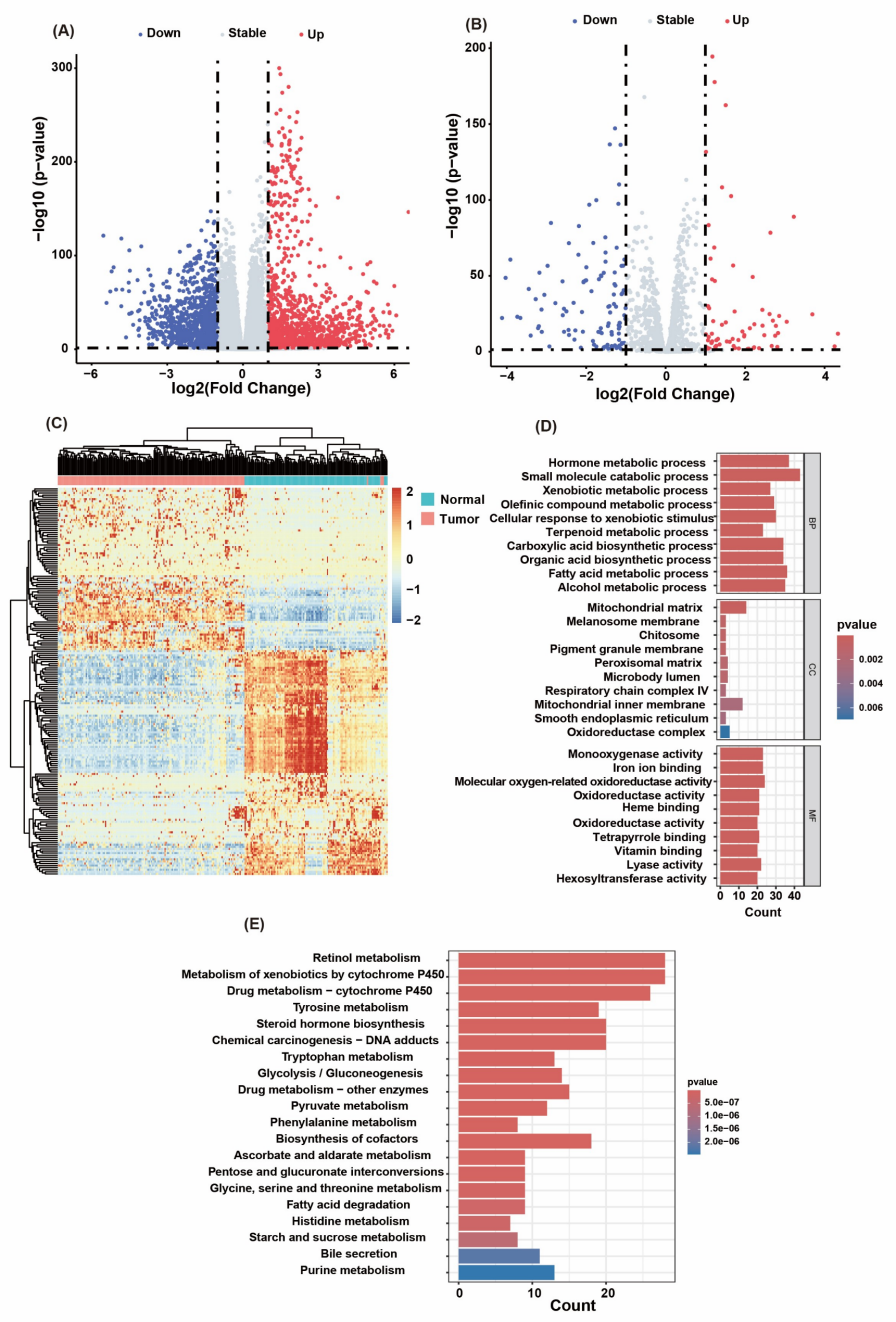
Metabolic gene differential expression analysis and functional enrichment analysis. (**A**) DEGs between TNBC patient and normal samples. (**B**) Identification of metabolism-related DEGs. (**C**) Expression patterns of metabolism-related DEGs in TNBC samples compared with normal samples, with red indicating higher expression and blue indicating lower expression. (**D**) GO enrichment analysis of metabolism-related DEGs. (**E**) KEGG enrichment analysis of metabolism-related DEGs

The workflow of this study is depicted in Fig. 1. A total of 158 TNBC patient samples and 114 normal samples were obtained from the TCGA. Utilizing the “DESeq2” R package, we identified 2,450 differentially expressed genes (DEGs) between TNBC and adjacent normal tissues with the criteria of|log2 (fold change)| > 1 and a false discovery rate (FDR) < 0.05, comprising 1,117 downregulated and 1,333 upregulated genes (Fig. 2A; Supplementary Table S2). From the KEGG database, we extracted 1,660 genes associated with 86 human metabolic pathways (Supplementary Table S1) [17]. Among these metabolic genes, 186 DEGs were identified, including 110 downregulated and 76 upregulated genes, which clearly separated TNBC samples from normal adjacent samples (Fig. 2B, C; Supplementary Table S3).

### Functional enrichment analysis of metabolic DEGs in TNBC reveals their involvement in key biological pathways and processes

To gain an in-depth understanding of the biological functions of metabolic DEGs, we conducted a comprehensive enrichment analysis. GO enrichment analysis revealed that these genes are involved primarily in pathways such as hormone metabolism, small molecule metabolism, xenobiotic metabolism, terpenoid metabolism, and fatty acid metabolism (Fig. 2D). KEGG enrichment analysis further refined the specific metabolic pathways in which these genes participate, including retinol metabolism, cytochrome P450 metabolism of xenobiotics, drug metabolism - cytochrome P450, steroid hormone biosynthesis, tyrosine metabolism, and tryptophan metabolism (Fig. 2E).

### Constructing a prognostic model for TNBC based on metabolic genes

**Fig. 3:**
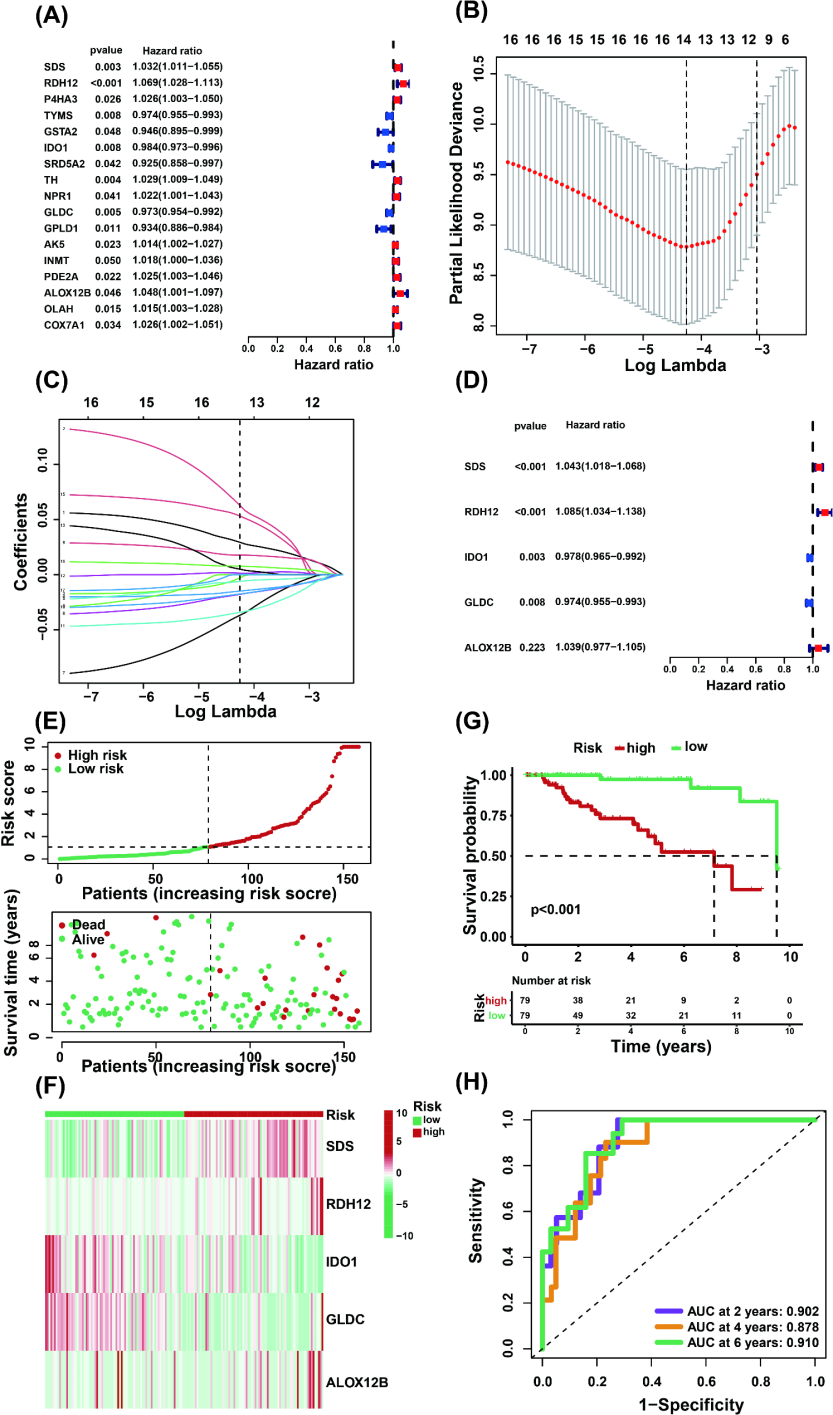
Development of a metabolism-related gene-based prognostic risk model in the TCGA cohort. (**A**) Univariate Cox regression analysis of differentially expressed metabolism-related genes. (Hazard Ratio (HR): Indicates the association between a gene and prognosis. HR > 1 means the gene is a risk factor, associated with worse prognosis; HR < 1 suggests means the gene is a protective factor, associated with better prognosis. *P* < 0.05 was considered statistically significant.). (**B**) Coefficient plot from the LASSO regression model (The left and right vertical lines respectively represent the λ value (Lambda Min) corresponding to the minimum cross - validation error and the simplest model (Lambda 1SE) within one standard error of the minimum MSE. Lambda Min was selected for subsequent analyses.). (**C**) Cross-validation for the selection of tuning parameters in the LASSO regression. (**D**) Multivariate Cox regression analysis revealed five key genes. (**E**) Distribution of patient survival status and survival time based on the prognostic model in the TCGA-TNBC cohort. (**F**) Expression profiles of five key genes in the prognostic model. (**G**) Survival analysis of TNBC patients of high- and low-risk group. (**H**) Validation of the predictive efficiency of the risk score via ROC curve analysis

Through univariate Cox regression analysis of metabolism-related DEGs, a total of 17 genes significantly associated with prognosis (*P* < 0.05) were identified, including 11 risk factors (Hazard Ratio (HR) > 1, *P* < 0.05): SDS, RDH12, P4HA3, TH, NPR1, AK5, INMT, PDE2A, ALOX12B, OLAH and COX7A1, and six protective factors (HR < 1, *P* < 0.05): TYMS, GSTA2, IDO1, SRD5A2, GLDC, and GPLD1 (Fig. 3A). Furthermore, LASSO regression analysis was conducted, revealing 14 genes (*P* < 0.05): SDS, RDH12, P4HA3, TYMS, GSTA2, IDO1, SRD5A2, TH, GLDC, GPLD1, AK5, INMT, ALOX12B and OLAH (Fig. 3B, C). Multivariate Cox regression analysis eventually identified five genes, among which three are potential risk genes (HR > 1, *P* < 0.05): SDS, RDH12 and ALOX12B and two are potential protective genes (HR < 1, *P* < 0.05): IDO1 and GLDC, which is suitable for constructing a prognostic model (Fig. 3D). On the basis of the above screening results, a prognostic index for TNBC samples was constructed with the following formula: risk score = 0.042232206 × SDS (expression level) + 0.081515243 × RDH12 (expression level) − 0.021784204 × IDO1 (expression level) − 0.02628676 × GLDC (expression level) + 0.038228584 × ALOX12B (expression level) [25–27]. To verify the accuracy of this model in predicting the prognosis of TNBC patients, 158 patients were divided into high- (*n* = 79) and low-risk (*n* = 79) groups on the basis of the median risk score threshold (Fig. 3E). Compared with the low-risk group, the high-risk group had a shorter survival time (*P* < 0.001, Fig. 3G). SDS, RDH12 and ALOX12B were highly expressed in the high-risk group samples, whereas IDO1 and GLDC were expressed at low levels (Fig. 3F). Time-dependent ROC analysis revealed that the accuracy of the OS prognosis was 0.902 at 2 years, 0.878 at 4 years, and 0.910 at 6 years (Fig. 3H). The results indicate that our metabolic gene model significantly predicts TNBC prognosis, as evidenced by high accuracy rates and robust statistical validation.

### Validation of a prognostic model for TNBC using GEO datasets

**Fig. 4:**
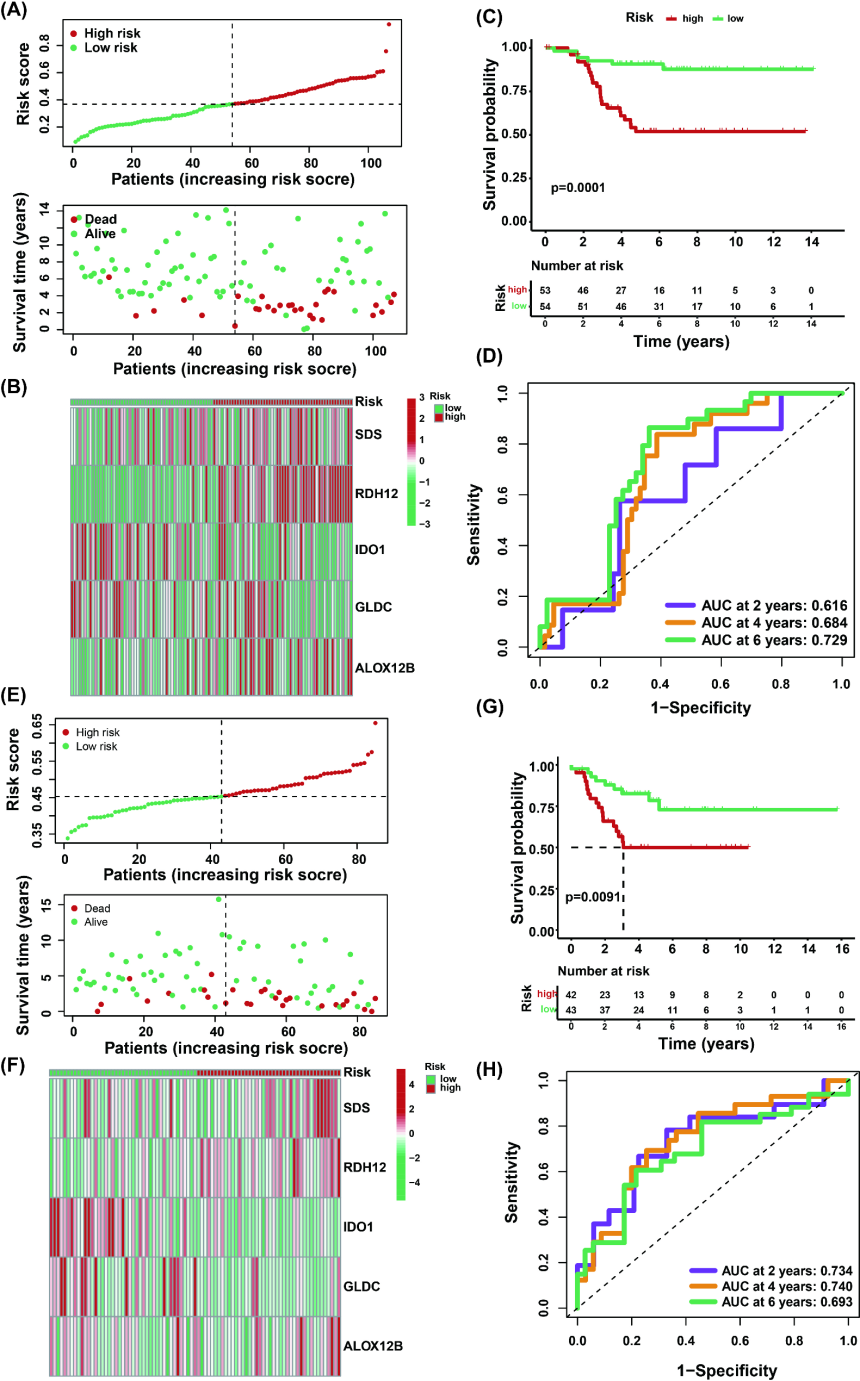
Validation of metabolism-related prognostic signatures in TNBC using two independent cohorts. (**A**) Distribution of patient survival status and survival time according to the risk score in the GSE58812 dataset. (**B**) Expression heatmap of five key genes in the low- and high-risk groups within the GSE58812 dataset. (**C**) Kaplan‒Meier survival curves for the low- and high-risk groups derived from the GSE58812 dataset. (**D**) Time-dependent ROC analysis of the 2-, 4-, and 6-year overall survival (OS) probabilities in the GSE58812 dataset. (**E**) Distribution of patient survival status and survival time according to the risk score in the GSE21653 dataset. (**F**) Expression heatmap of five key genes in the low- and high-risk groups within the GSE21653 dataset. (**G**) Kaplan-Meier survival curves for the low- and high-risk groups derived from the GSE21653 dataset. (**H**) Time-dependent ROC analysis of the 2-, 4-, and 6-year overall survival (OS) probabilities in the GSE21653 dataset

To validate the generalizability of the established prognostic model, we performed external validation using two independent TNBC cohorts (GSE58812 and GSE21653) retrieved from GEO. Applying the risk score formula derived from the training cohort, patients were stratified into distinct low- and high-risk subgroups according to the median cutoff. Strikingly, the low-risk group exhibited significantly longer median overall survival compared to high-risk counterparts across both validation cohorts (Fig. 4A, E). Importantly, the expression patterns of model-defining genes showed high concordance with TCGA-TNBC profiles (Fig. 4B, F), reinforcing their biological relevance as prognostic biomarkers in TNBC pathophysiology. The clinical utility of this stratification was further corroborated by Kaplan-Meier analysis, which revealed markedly poorer outcomes in high-risk patients (*P* < 0.001; Fig. 4C, G). Temporal discrimination accuracy was quantified through time-specific ROC analysis, yielding AUC values of 0.616 (2-year), 0.684 (4-year), and 0.729 (6-year) in GSE58812, and 0.734, 0.740, and 0.693 respectively in GSE21653 (Fig. 4D, H). These metabolism-associated gene signatures not only provide a novel prognostic framework for TNBC but also inform potential therapeutic targeting strategies. Multivariate Cox regression confirmed the risk score as an independent prognostic factor (AUC = 0.889), outperforming conventional clinicopathological parameters (Supplementary Fig. 1).

### Functional enrichment analysis of differential gene expression based on the risk model

**Fig. 5:**
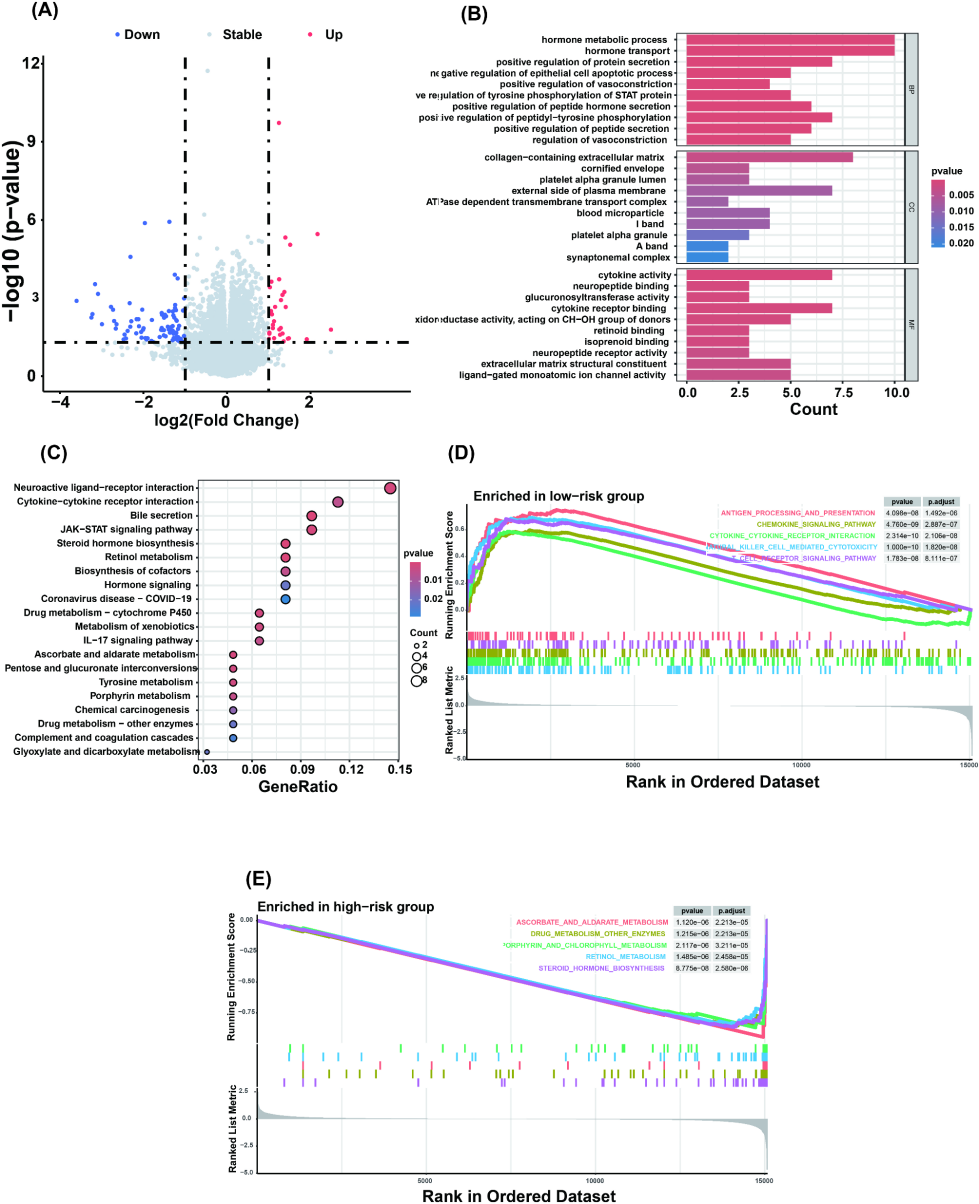
Differentially expressed genes and functional enrichment analysis between the low- and high-risk groups. (**A**) Volcano plot of DEGs between the low- and high-risk groups in the TCGA cohort. (**B**) Bar chart of the results of the GO enrichment analysis, which revealed the significance of the biological processes. (**C**) KEGG enrichment analysis, bubble size indicates the number of enriched genes, and color intensity indicates the significance of the difference (q value: adjusted *P* value). (**D**) The top 5 significantly upregulated pathways identified via GSEA of the low-risk group samples. (**E**) The top 5 significantly upregulated pathways identified via GSEA of the high-risk group samples

Using the “DESeq2” R package, we identified 120 DEGs between the high- and low-risk groups, with 30 genes upregulated and 90 downregulated in the low-risk group (Fig. 5A; Supplementary Table S4). GO enrichment analysis revealed significant differences in processes such as hormone metabolism and hormone transport between the high- and low-risk groups (Fig. 5B). KEGG enrichment analysis demonstrated that the DEGs were significantly enriched in ​cytokine-cytokine receptor interaction​, ​steroid hormone biosynthesis​, and ​drug metabolism pathways, with notable involvement of the JAK-STAT signaling pathway and retinol metabolism. These findings highlight potential interplay between immune dysregulation (e.g., IL-17 signaling), metabolic reprogramming, and xenobiotic detoxification in TNBC progression (Fig. 5C). Further GSEA revealed pronounced enrichment of immunomodulatory pathways in the low-risk group, most notably ​natural killer cell-mediated cytotoxicity, ​cytokine-cytokine receptor interaction​, and ​antigen processing/presentation​. Additional significant pathways included ​T cell receptor signaling​ and ​chemokine signaling​, collectively indicating enhanced anti-tumor immunity through NK cell activation, T cell priming, and chemokine-guided leukocyte infiltration (Fig. 5D). GSEA analysis revealed that metabolic pathways in high-risk TNBC patients were significantly enriched in steroid hormone biosynthesis, ascorbate metabolism, cytochrome P450/drug metabolism, while porphyrin metabolism and retinol metabolism pathways also exhibited activation (Fig. 5E). These metabolic reprogramming features may influence chemotherapy resistance.

### Comparison of somatic mutation profiles and tumor mutational burden between the high- and low-risk groups

**Fig. 6:**
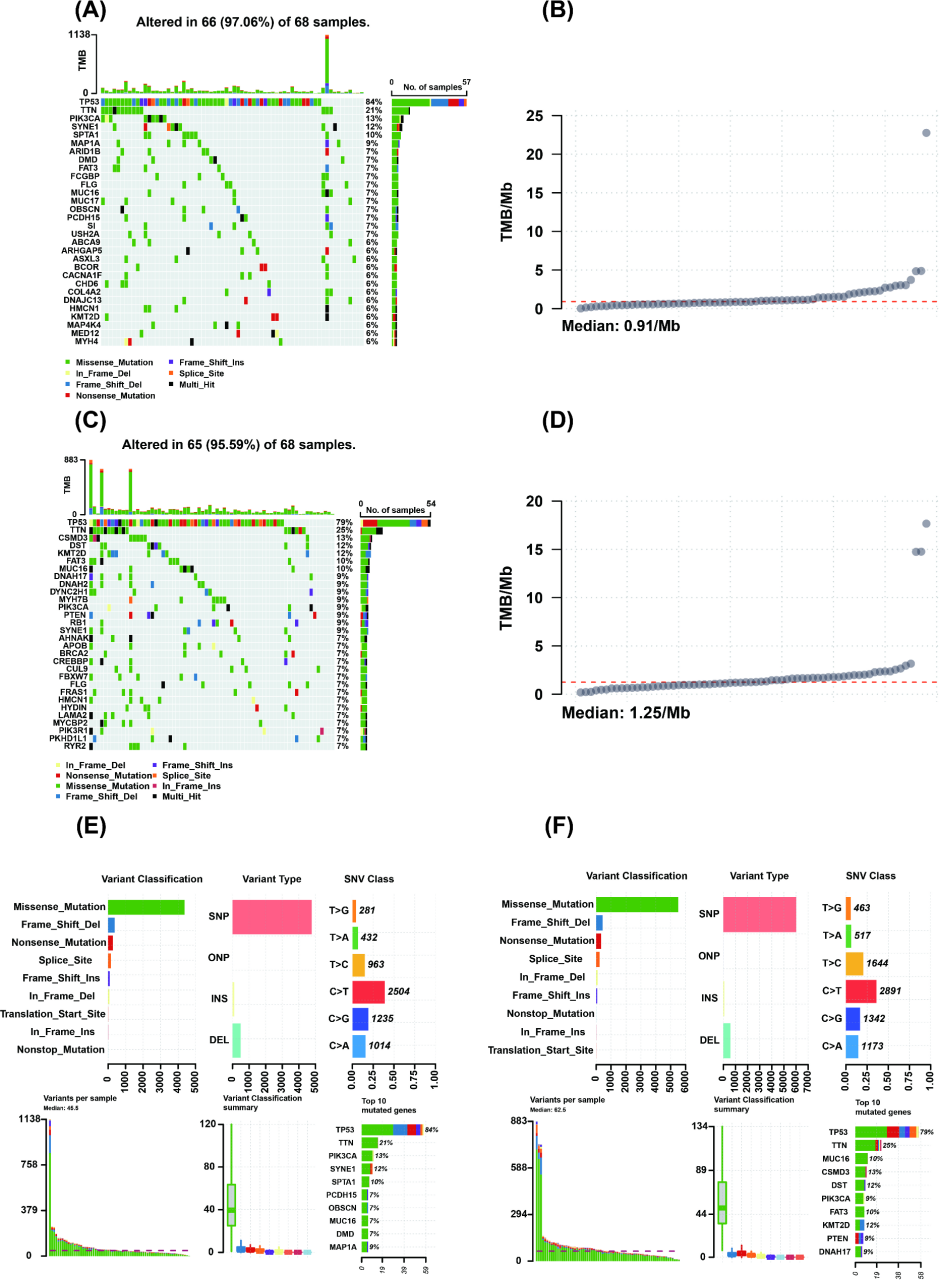
Mutational spectra and burden in the high- and low-risk groups. (**A**) Mutation waterfall plot for samples in the high-risk group. (**B**) Mutational burden in samples from the high-risk group. (**C**) Mutation waterfall plot for samples in the low-risk group. (**D**) Mutational burden in samples from the low-risk group. (**E**) and (**F**) Detailed mutational profiles of samples from the high- and low-risk groups

We analyzed the somatic mutation profiles and tumor mutational burden (TMB) in samples from the high- and low-risk groups. The mutation rates were comparable between the two group samples, with 97.06% (66/68) in the high-risk group and 95.59% (65/68) in the low-risk group (Fig. 6A, C). The median TMB was 0.91 mutations/Mb in the high-risk group and 1.25 mutations/Mb in the low-risk group, suggesting a significantly higher TMB in the latter. This elevated TMB may be associated with improved responsiveness to immunotherapy, highlighting its potential as a predictive biomarker for treatment outcomes (Fig. 6B, D). We conducted an in-depth analysis of the somatic mutational profiles of samples from both the high- and low-risk groups to explore the similarities and differences in mutational characteristics. In terms of mutation types, both groups exhibited a high degree of similarity in mutational patterns. Missense mutations accounted for the highest proportion, followed by frameshift deletions and nonsense mutations. Regarding single nucleotide variant (SNV) classification, C > T and T > C base substitutions predominated in both groups. Single nucleotide polymorphisms (SNPs) represented the most prevalent variant type in both cohorts, with deletion mutations being the second most common alteration. While single nucleotide variant (SNV) classification and mutational spectrum patterns were comparable between groups, the high-risk cohort exhibited a significantly reduced total mutation burden (mean 45.5 mutations/sample) compared to the low-risk group (mean 62.5 mutations/sample; *P* < 0.05). This discrepancy may reflect the notably higher TMB observed in the low-risk cohort. We further analyzed the top mutated genes in both the high- and low-risk groups and found that both groups shared similar high-frequency mutated genes, including TP53, TTN, PIK3CA, etc. However, distinct mutation profiles were observed between risk groups: genes including CSMD3, DST, and KMT2D exhibited higher mutational prevalence in the low-risk cohort, while SYNE1, SPTA1, and PCDH15 demonstrated elevated mutation frequencies in the high-risk group (Fig. 6E, F). Notably, the TP53 gene had a high mutation frequency in both groups, at 84% and 79%, emphasizing the crucial role of TP53 in tumorigenesis. In summary, our analysis revealed that while both high- and low-risk groups share similar mutation profiles and high-frequency mutated genes, distinct mutational patterns and a higher TMB in the low-risk group may have significant implications for treatment responsiveness and outcomes.

### Assessment of tumor immune evasion, the immunotherapy response, and drug sensitivity in the high- and low-risk groups

**Fig. 7:**
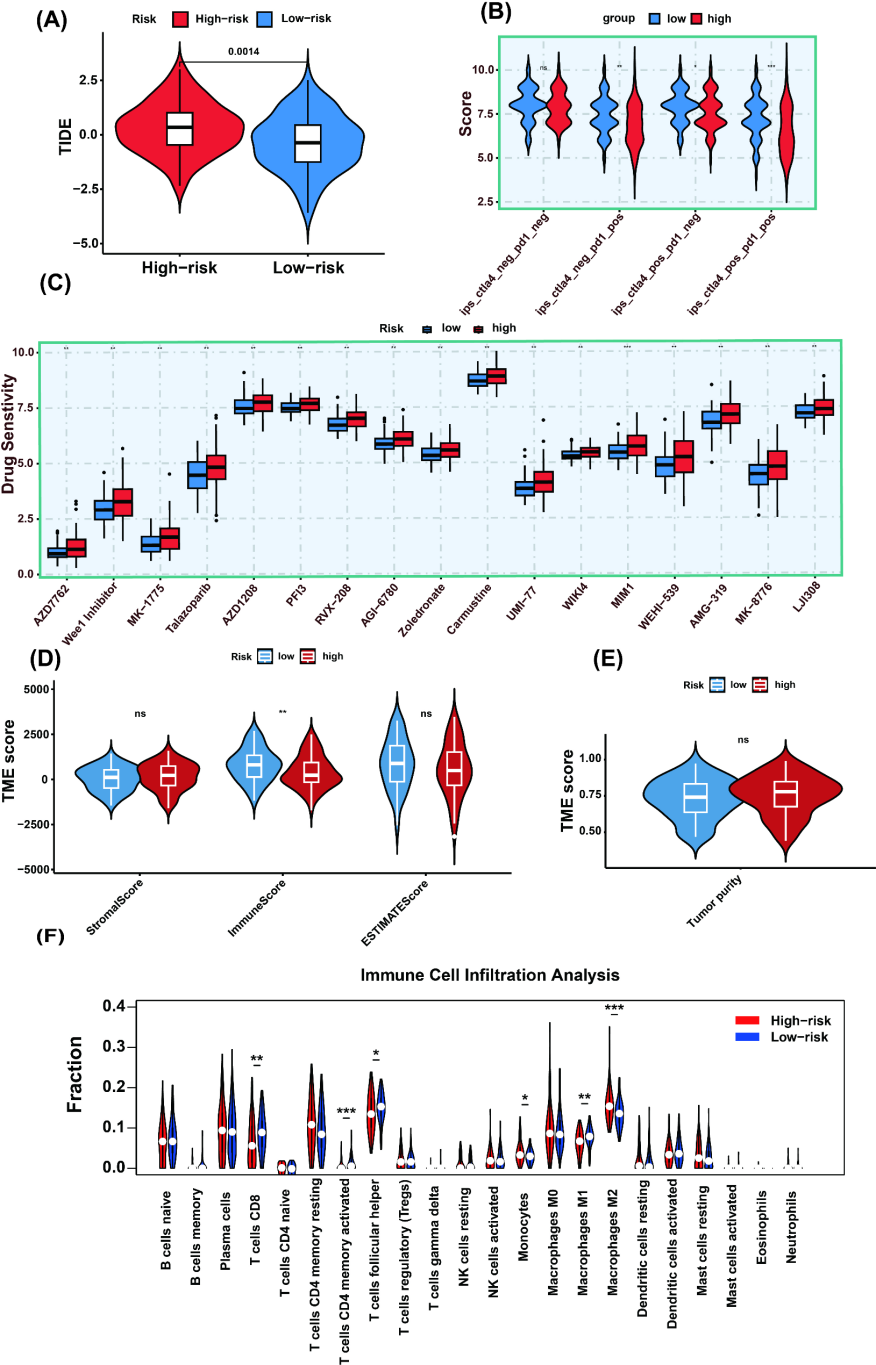
Immunotherapy response, drug treatment sensitivity, and immune response in high- and low-risk groups. (**A**) TIDE score. (**B**) Response to immunotherapy. (**C**) Sensitivity to drugs. (**D**) Stromal score, Immune score and ESTIMATE score. (**E**) Tumor purity. (**F**) Immune infiltration levels in high- and low-risk groups of TNBC patients determined via CIBERSORT

TIDE stands for tumor immune dysfunction, and exclusion is a metric based on the gene expression profile of tumor samples used to evaluate the potential for tumor immune evasion. Analysis revealed that samples from the high-risk group had higher TIDE scores, suggesting a greater likelihood of immune evasion (*P* = 0.0014, Fig. 7A). When comparing immune therapy responses between high- and low-risk groups, the low-risk group exhibited significantly elevated scores for ips_ctal4_neg_pd1_pos, ips_ctal4_pos_pd1_neg, and ips_ctal4_pos_pd1_pos, collectively suggesting enhanced T-cell activation and tumoricidal immunity, which may confer superior clinical sensitivity to PD-1/CTLA-4 checkpoint blockade therapies. However, there was no significant difference in scores for ips_ctal4_neg_pd1_neg (Fig. 7B). Drug sensitivity profiling between risk cohorts identified 17 pharmacologic agents with significant differential sensitivity (*P* < 0.05). Notably, enhanced therapeutic responses to AZD7762, WEE1 inhibitor, MK-1775, talazoparib, AZD1208, PF13, RVX-208, AGI-6780, zoledronate, carmustine, UMI-77, WIKI4, MIM1, WEHI-539, AMG-319, MK-8776, and LJ308 were observed in the high-risk group, suggesting clinically actionable targets for precision oncology strategies in these patients (Fig. 7C).

### Analysis of the tumor microenvironment and immune cell infiltration in high- and low-risk group samples

To assess the differences in the tumor microenvironment (TME) between the high- and low-risk groups, we calculated stromal scores, immune scores, ESTIMATE scores, and tumor purity for all samples. The analysis revealed that ​high-risk group samples exhibited significantly lower immune scores​ compared to the low-risk group (*P* < 0.01, Fig. 7D). However, ​no significant differences​ were observed between the two groups in stromal scores, ESTIMATE scores (Fig. 7D), or tumor purity (Fig. 7E). Further analysis of immune cell infiltration by CIBERSORT in high- and low-risk groups demonstrated that the low-risk cohort exhibited significantly elevated levels of CD8 + T cells, activated CD4 + memory T cells, follicular helper T cells, and M1 macrophages compared to the high-risk group. Conversely, monocyte counts and M2 macrophage infiltration were markedly reduced in low-risk patients, which may correlate with better immune surveillance and antitumor capabilities (Fig. 7F). The ssGSEA results demonstrated that low-risk group samples exhibited significantly elevated scores in multiple activated immune cell populations, including activated B cells, activated CD4 + T cells, activated CD8 + T cells, activated dendritic cells, immature B cells, Type-1 T helper cells and Type-2 T helper cells (Supplementary Fig. 2). These findings suggest enhanced immune cell activation and functional engagement within the low-risk group.

### IDO1 plays a critical role in the metabolic-related prognostic model of TNBC

**Fig. 8:**
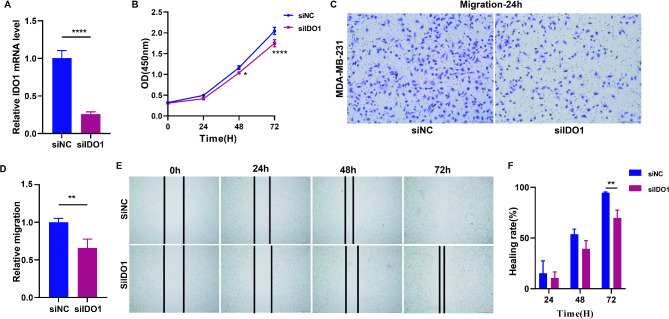
Effect of IDO1 silencing on the growth and migration of breast cancer cells. (**A**) RT-qPCR detection of IDO1 mRNA knockdown. (**B**) Assessment of the growth curve of IDO1-knockdown MDA-MB-231 cells through the CCK8 assay. (**C**) - (**D**) Transwell assay showing the effect of IDO1 silencing on cell migration. (**E**) - (**F**) Scratch assay showing the effect of IDO1 silencing on cell migration

Based on the TCGA-TNBC dataset analysis, four out of the five model genes (SDS, RDH12, IDO1, and GLDC) demonstrated significantly elevated expression levels in breast cancer samples (Supplementary Fig. 3A - E). The survival analysis of TNBC patients based on the median expression values of five model genes demonstrated that SDS, IDO1, and GLDC genes significantly influenced patient prognosis (Supplementary Fig. 4A - E). These findings highlight their potential roles as prognostic biomarkers or therapeutic targets in TNBC management. We further validated the expression of three key genes in breast cancer cell lines (MDA-MB-231 and MCF-7) and normal breast cells (MCF-10 A) via RT-qPCR and found that IDO1 is significantly overexpressed in MDA-MB-231 cells. Meanwhile, we found that the GLDC gene is significantly highly expressed in both MCF-7 and MDA-MB-231 (Supplementary Fig. 3F - H). The expression trends of these genes in two breast cancer cell lines (MDA-MB-231 and MCF-7) were further validated in cancer cell lines from the Cancer Cell Line Encyclopedia (CCLE) database, confirming consistent gene expression patterns across different cell models (Supplementary Fig. 3I). Given the significant higher of IDO1 in the TNBC cell line MDA-MB-231, we posit that IDO1 likely plays a crucial role in TNBC progression. Consequently, IDO1 was prioritized for subsequent investigation. Further analysis identified IDO1 as a key gene within the metabolism-related prognostic model, prompting additional in vitro experimentation. Silencing IDO1 via IDO1-specific siRNA transfection resulted in a reduction in IDO1 mRNA in MDA-MB-231 cells (Fig. 8A). The CCK8 proliferation assay revealed significant inhibition of growth in the IDO1-depleted MDA-MB-231 cell line (Fig. 8B). Additionally, the downregulation of IDO1 led to the suppression of breast cancer cell migration, as evidenced by the Transwell migration assay (Fig. 8C, D) and the scratch wound healing assay (Fig. 8E, F). Thus, IDO1 can promote the proliferation and migration of breast cancer cells, thereby playing a pivotal role in the development of TNBC, which further substantiates its critical function in TNBC.

## Discussion

TNBC is a distinct subtype of breast cancer characterized by a poor prognosis and limited treatment options. Therefore, identifying new biomarkers and therapeutic targets is crucial [1–4]. Metabolic reprogramming is a hallmark of cancer, influencing tumor growth, the tumor microenvironment (TME), and interactions between immune cells [16]. In TNBC, tumor cells exhibit metabolic characteristics distinct from those of normal cells, including unique patterns of glucose, fatty acid, and amino acid metabolism [13]. Therefore, we explored the clinical significance of metabolism-related genes in breast cancer. Initially, we identified a total of 186 DEGs associated with metabolism (Fig. 2B - C). These genes are involved in various biological processes and pathways, including hormone metabolism, terpenoid metabolism, fatty acid metabolism, retinol metabolism, steroid hormone biosynthesis, tryptophan metabolism, etc. (Fig. 2D - E). These findings indicate that these biological processes and pathways play significant roles in TNBC. Furthermore, after a series of rigorous screenings, key metabolic genes with differential expression and prognostic correlations, including SDS, RDH12, IDO1, GLDC, and ALOX12B, were identified in TNBC and shown to serve as promising prognostic biomarkers for TNBC. On the basis of these genes, we constructed a risk model in the TCGA-TNBC dataset, calculated the risk scores, investigated their prognostic and predictive values, as well as their strong associations with TNBC (Fig. 3A - H), and further validated their good performance in other external cohorts (GSE58812 and GSE21653) (Fig. 4A - H). Survival analysis demonstrated a distinct separation of Kaplan-Meier curves between high- and low-risk subgroups stratified by prognostic scores within the high-risk cohort (Figs. 3G and 4C and G). Our study revealed that patients in the high-risk group had poorer clinical conditions and survival outcomes than did those in the low-risk group. Additionally, treatment failure is more likely to be observed in TNBC patients in the high-risk group.

Gene differential analysis and functional enrichment analysis of the high- and low-risk groups revealed significantly enriched biological processes and signaling pathways, providing a new perspective for understanding the molecular mechanisms of TNBC. The analysis revealed that genes involved in hormone metabolism and transport processes exhibited significant upregulation in the high-risk group, while concurrent immune dysregulation was observed within this cohort. (Fig. 5B, C). This finding is consistent with the absence of hormone receptor expression in TNBC, suggesting that these pathways may influence tumor progression through noncanonical routes. Furthermore, the activation of the steroid hormone biosynthesis pathway may be closely related to the proliferation and survival of tumor cells [34]. Similarly, we also found significant enrichment in the metabolic pathways of cytochrome P450 drug metabolism and other drug metabolism enzymes in high-risk patients, which may be related to their metabolism of chemotherapeutic drugs and drug resistance (Fig. 5E). Retinol plays a key role in cell differentiation and proliferation [35]. Patients in the high-risk group were significantly enriched in the retinol metabolism pathway, which may be associated with abnormal cell differentiation and proliferation (Fig. 5C, E). Concurrently, we also observed significant enrichment in the cytokine-cytokine receptor interaction pathway among patients in the low-risk group (Fig. 5C, D). These findings suggest that the immune system may play a complex role in the progression of TNBC, particularly in regulating inflammatory responses and immune evasion within the TME. Antigen processing and presentation were also found to be significantly enriched in low-risk patients, possibly indicating better immune recognition and response (Fig. 5D) [36]. Natural killer (NK) cells play crucial roles in antitumor immunity [37]. Our analysis revealed that patients in the low-risk group were significantly enriched in NK cell-mediated cytotoxicity pathways, which may be related to improved immune surveillance and tumor control capabilities (Fig. 5D).

This study revealed a high prevalence of somatic mutations (> 95%) in TNBC patients, highlighting the genomic instability characteristic of TNBC patients. This genomic feature may significantly contribute to the tumor’s highly aggressive behavior and poor therapeutic response observed in clinical settings. However, we found that low-risk group presented a significantly greater TMB than did the high-risk group patients (Fig. 6A - D). This finding is in accordance with previous research, which suggests that a higher TMB may be positively correlated with responsiveness to immunotherapy [38]. Consequently, patients with low-risk TNBC may possess a potential therapeutic advantage. Both high- and low-risk groups exhibited remarkable concordance in somatic mutation profiles, with missense mutations constituting the predominant variant type, followed sequentially by frameshift deletions and nonsense mutations (Fig. 6E, F). This striking similarity in mutational landscape architecture suggests potential convergence in tumorigenic pathways between the two risk stratifications [38]. Interestingly, our analysis revealed a lower somatic mutation rate in the high-risk cohort compared to the low-risk group, which aligns with the observation of a significantly higher TMB in the latter (Fig. 6E, F). This inverse correlation between mutation rate and risk stratification may suggest a potential association with improved prognosis in the low-risk cohort, possibly mediated by enhanced immune surveillance or differential genomic instability mechanisms. In terms of frequently mutated genes, the high mutation frequency of the TP53 gene in both groups further underscores its central role in tumorigenesis [39]. Our analysis revealed that high- and low-risk groups shared recurrently mutated genes including TP53, TTN, and PIK3CA, suggesting their potential roles as core drivers in TNBC tumorigenesis. Notably, distinct mutational patterns emerged between subgroups: genes such as CSMD3, DST, and KMT2D demonstrated higher mutational frequency in the low-risk cohort, while SYNE1, SPTA1, and PCDH15 were significantly enriched in the high-risk group (Fig. 6E, F). These differentially mutated genes may serve as subgroup-specific biomarkers and represent promising therapeutic targets, highlighting the molecular heterogeneity underlying TNBC progression. Additionally, these findings may contribute to the identification of new therapeutic targets, offering more precise treatment plans for TNBC patients.

The tumor immune evasion potential in TNBC patients was evaluated using the TIDE scoring system. Analysis demonstrated significantly elevated TIDE scores in the high-risk cohort compared to low-risk group (Fig. 7A), suggesting enhanced immune evasion mechanisms in high-risk group patients that may facilitate resistance to host immune surveillance. This differential immune escape capacity between risk groups highlights distinct biological behaviors in TNBC progression and warrants further investigation into targeted immunotherapeutic strategies. Consistent with the TIDE analysis, low-risk patients showed significantly higher scores for both ips_ctal4_neg_pd1_pos and ips_ctal4_pos_pd1_pos compared to high-risk group patients (Fig. 7B). These findings suggest enhanced responsiveness to anti-PD-1 immunotherapy in the low-risk cohort, highlighting the potential clinical utility of these biomarkers in personalizing immunotherapy regimens. Pharmacological sensitivity profiling demonstrated statistically significant differences (*P* < 0.05) in drug responses to 17 therapeutic agents between the two cohorts (Fig. 7C). Of particular clinical relevance, the high-risk cohort exhibited enhanced sensitivity to targeted agents including WEE1 inhibitors (regulators of G2/M checkpoint) and MK-1775 (a selective small-molecule WEE1 inhibitor). This differential sensitivity profile may be mechanistically linked to the high prevalence of TP53 mutations (84% mutation rate) and compromised DNA damage repair capacity observed in the high-risk group. Preclinical evidence suggests that WEE1 inhibitors exert selective cytotoxicity in TP53-deficient tumors through abrogation of G2/M checkpoint control, thereby forcing premature entry of DNA-damaged cells into mitosis and subsequent induction of mitotic catastrophe [40]. This study suggests that specific drugs hold potential clinical application prospects in the treatment of TNBC. The risk stratification model constructed based on tumor gene expression profiles provides critical evidence for precision medicine in TNBC: high-risk patients may require intensive therapeutic regimens guided by drug sensitivity profiles, while low-risk patients are more likely to benefit from immunotherapy, establishing a theoretical foundation for personalized treatment strategies. Future research should focus on elucidating the molecular regulatory networks between TIDE scores and the tumor immune microenvironment, accelerating the translation of relevant biomarkers into clinical prediction tools. Concurrently, in-depth exploration of the molecular mechanisms underlying drug sensitivity heterogeneity may reveal novel therapeutic targets. It is noteworthy that current drug sensitivity data predominantly originate from bioinformatics predictions, necessitating validation through in vitro and in vivo experimental models as well as multicenter clinical studies to confirm clinical applicability. For candidate drugs with incompletely characterized mechanisms (e.g., AGI-6780, RVX-208), molecular pathway investigations are recommended to clarify their biological associations with risk stratification. Given the marked intratumoral heterogeneity of TNBC, expanding cohort sizes and integrating multi-omics analyses will be essential pathways for verifying the generalizability of research findings.

An in-depth characterization of the tumor microenvironment and immune infiltration patterns was performed in high-risk versus low-risk TNBC cohorts. Quantitative assessment demonstrated a markedly reduced tumor immune score in high-risk patients compared to low-risk counterparts (Fig. 7D), indicative of compromised anti-tumor immunity that potentially contributes to enhanced tumor aggressiveness and unfavorable clinical outcomes. Notably, while ESTIMATE composite metrics including stromal components and tumor purity showed comparable profiles between groups (Fig. 7D, E), differential immune cell infiltration patterns emerged through detailed subset analysis. The low-risk cohort exhibited substantially elevated infiltration levels of key effector populations, including cytotoxic CD8 + T lymphocytes, activated CD4 + memory T cells, follicular helper T cells, and pro-inflammatory M1-polarized macrophages (Fig. 7F). These findings collectively highlight the critical association between specific immune contextures and TNBC risk stratification, suggesting that immune effector depletion rather than global microenvironmental alterations may drive prognostic differences. These types of immune cells are associated with immune activation and antitumor capabilities, and their higher expression in the low-risk group may be correlated with better immune surveillance and antitumor responses. In contrast, the levels of monocyte counts and M2 macrophage infiltration were significantly greater in the high-risk group samples (Fig. 7F), suggesting a potential association with immune evasion by the tumor. The results of ssGSEA further corroborated the aforementioned findings (Supplementary Fig. 2), indicating a greater degree of immune cell activation and involvement in the low-risk group samples. These discoveries underscore the pivotal role of immune cells in the tumor microenvironment of TNBC and may provide a basis for the personalization of immunotherapy approaches. The tumor microenvironment of the low-risk group may be more conducive to the activation of immune cells and antitumor responses, which could be the basis for its better prognosis and responsiveness to immunotherapy. Future research will need to further explore the specific roles of these immune cells in the development of TNBC, as well as how to utilize this information to improve treatment strategies.

We conducted an in-depth analysis of metabolism-related genes in TNBC, with particular attention given to the potential role of the IDO1 gene. IDO1 is a key enzyme in the tryptophan metabolic pathway, and its high expression in the tumor microenvironment is closely associated with immune evasion [41]. By regulating tryptophan metabolism, IDO1 can induce T-cell cycle arrest or autophagy and inhibit the mTOR signaling pathway, thereby reducing T-cell activity [42]. Moreover, the metabolic byproducts of tryptophan bind to the aryl hydrocarbon receptor, counteracting signals that activate T cells and facilitating the immune evasion of tumor cells [43]. Through analysis of the TCGA-TNBC database, we found that the expression levels of IDO1 in breast cancer tissue were significantly greater than those in normal breast tissue (Supplementary Fig. 3C). This finding is consistent with our RT-qPCR results, where high expression of IDO1 was particularly prominent in the MDA-MB-231 cell line (Supplementary Fig. 3G, I), suggesting that IDO1 may play a pivotal role in the progression of TNBC. These results highlight the crucial role of IDO1 in the proliferation and migration of TNBC cells, thereby exerting a central influence on the development of TNBC. However, in our study, the expression levels of IDO1 were generally lower in high-risk group patients than in low-risk group patients (Figs. 3F and 4B and F), indicating that the role of IDO1 in the tumor microenvironment may be highly complex, potentially exerting different functions in various tumor microenvironments or among different patients. In some cases, high expression of IDO1 might be associated with immune evasion and tumor progression, whereas in other scenarios, it could be linked to immune activation and antitumor responses. The functions of IDO1 are likely related to its role in modulating immune responses and the tumor microenvironment, providing strong evidence that IDO1 is a therapeutic target for TNBC. Our findings not only offer new insights into the biological significance of IDO1 in TNBC but also provide a scientific basis for the development of novel treatment strategies targeting IDO1. Future research should further explore the molecular mechanisms of IDO1 in TNBC and how to effectively translate these discoveries into clinical applications. Additionally, the development and testing of IDO1 inhibitors could provide new treatment options for TNBC patients, especially when conventional therapeutic approaches have limited efficacy. Our study provides a significant theoretical foundation for the use of IDO1 as a potential therapeutic target for TNBC and may have a profound impact on treatment strategies for TNBC.

## Conclusion

In summary, this study emphasized the key role of metabolism-related genes in the prognosis of TNBC patients and established a robust prognostic model that integrates these genes. The identification of 186 differentially expressed metabolic genes not only highlights the significant changes in metabolic pathways associated with TNBC but also provides a foundation for future research targeting therapies. Furthermore, the correlation between the risk scoring model and clinical parameters enhances the accuracy of prognostic assessment, paving the way for personalized treatment strategies. The findings regarding immune evasion and drug sensitivity further underscore the potential for tailoring treatment approaches on the basis of individual patient profiles. However, the limitations of sample size and the necessity for experimental validation must be addressed in future studies to strengthen the applicability of these results in clinical settings. Overall, this research provides valuable insights into the molecular basis of TNBC and lays the groundwork for advancing precision medicine in this challenging subtype of cancer.

## Electronic supplementary material

Below is the link to the electronic supplementary material.

Supplementary Material 1

Supplementary Material 2

Supplementary Material 3

Supplementary Material 4

Supplementary Material 5

## Data Availability

The datasets analysed during the current study are available in the TCGA repository at https://portal.gdc.cancer.gov/ and GEO repository at https://www.ncbi.nlm.nih.gov/geo/ with accession numbers GSE58812 and GSE21653.

## Abbreviations

TNBC: Triple-negative breast cancer
TCGA: The Cancer Genome Atlas
GEO: Gene Expression Omnibus
ER: Expression of estrogen receptors
PR: Progesterone receptors
HER2: Human epidermal growth factor receptor 2
PARP: Poly (ADP-ribose) polymerase
DEGs: Differentially expressed genes
KEGG: Kyoto Encyclopedia of Genes and Genomes
GO: Gene Ontology
GSEA: Gene Set Enrichment Analysis
LASSO: Least absolute shrinkage and selection operator
ROC: Receiver operating characteristic
SNPs: Single nucleotide polymorphisms
IPS: Immunophenotype scores
TCIA: The Cancer Immunome Atlas
GDSC: Genomics of Drug Sensitivity in Cancer
OS: Overall survival
TIDE: Tumor Immune Dysfunction and Exclusion
TME: Tumor microenvironment
NK: Natural killer
Tfh: Follicular helper T cells
HR: Hazard Ratio

## References

[1] Carey L, Winer E, Viale G, Cameron D, Gianni L. Triple-negative breast cancer: disease entity or title of convenience? Nat Rev Clin Oncol. 2010;7(12):683–92. 10.1038/nrclinonc.2010.154. 20877296 10.1038/nrclinonc.2010.154

[2] Dent R, Trudeau M, Pritchard KI, Hanna WM, Kahn HK, Sawka CA, Lickley LA, Rawlinson E, Sun P, Narod SA. Triple-negative breast cancer: clinical features and patterns of recurrence. Clin Cancer Res. 2007;13(15 Pt 1):4429–34. 10.1158/1078-0432.Ccr-06-3045. 17671126 10.1158/1078-0432.CCR-06-3045

[3] Venkitaraman R. Triple-negative/basal-like breast cancer: clinical, pathologic and molecular features. Expert Rev Anticancer Ther. 2010;10(2):199–207. 10.1586/era.09.189. 20131996 10.1586/era.09.189

[4] Yin WJ, Lu JS, Di GH, Lin YP, Zhou LH, Liu GY, Wu J, Shen KW, Han QX, Shen ZZ, et al. Clinicopathological features of the triple-negative tumors in Chinese breast cancer patients. Breast Cancer Res Treat. 2009;115(2):325–33. 10.1007/s10549-008-0096-0. 18563552 10.1007/s10549-008-0096-0

[5] Dignam JJ, Dukic V, Anderson SJ, Mamounas EP, Wickerham DL, Wolmark N. Hazard of recurrence and adjuvant treatment effects over time in lymph node-negative breast cancer. Breast Cancer Res Treat. 2009;116(3):595–602. 10.1007/s10549-008-0200-5. 18830816 10.1007/s10549-008-0200-5 PMC2711214

[6] Jiang YZ, Ma D, Suo C, Shi J, Xue M, Hu X, Xiao Y, Yu KD, Liu YR, Yu Y, et al. Genomic and transcriptomic landscape of Triple-Negative breast cancers: subtypes and treatment strategies. Cancer Cell. 2019;35(3):428–40. 10.1016/j.ccell.2019.02.001. 30853353 10.1016/j.ccell.2019.02.001

[7] Lehmann BD, Bauer JA, Chen X, Sanders ME, Chakravarthy AB, Shyr Y, Pietenpol JA. Identification of human triple-negative breast cancer subtypes and preclinical models for selection of targeted therapies. J Clin Invest. 2011;121(7):2750–67. 10.1172/jci45014. 21633166 10.1172/JCI45014 PMC3127435

[8] Lehmann BD, Jovanović B, Chen X, Estrada MV, Johnson KN, Shyr Y, Moses HL, Sanders ME, Pietenpol JA. Refinement of Triple-Negative breast cancer molecular subtypes: implications for neoadjuvant chemotherapy selection. PLoS ONE. 2016;11(6):e0157368. 10.1371/journal.pone.0157368. 27310713 10.1371/journal.pone.0157368 PMC4911051

[9] Xiao Y, Ma D, Zhao S, Suo C, Shi J, Xue MZ, Ruan M, Wang H, Zhao J, Li Q, et al. Multi-Omics profiling reveals distinct microenvironment characterization and suggests immune escape mechanisms of Triple-Negative breast cancer. Clin Cancer Res. 2019;25(16):5002–14. 10.1158/1078-0432.Ccr-18-3524. 30837276 10.1158/1078-0432.CCR-18-3524

[10] Robson M, Im SA, Senkus E, Xu B, Domchek SM, Masuda N, Delaloge S, Li W, Tung N, Armstrong A, et al. Olaparib for metastatic breast cancer in patients with a germline BRCA mutation. N Engl J Med. 2017;377(6):523–33. 10.1056/NEJMoa1706450. 28578601 10.1056/NEJMoa1706450

[11] Schmid P, Turner NC, Barrios CH, Isakoff SJ, Kim SB, Sablin MP, Saji S, Savas P, Vidal GA, Oliveira M, et al. First-Line Ipatasertib, Atezolizumab, and taxane triplet for metastatic Triple-Negative breast cancer: clinical and biomarker results. Clin Cancer Res. 2024;30(4):767–78. 10.1158/1078-0432.Ccr-23-2084. 38060199 10.1158/1078-0432.CCR-23-2084 PMC10870115

[12] Bianchini G, Balko JM, Mayer IA, Sanders ME, Gianni L. Triple-negative breast cancer: challenges and opportunities of a heterogeneous disease. Nat Rev Clin Oncol. 2016;13(11):674–90. 10.1038/nrclinonc.2016.66. 27184417 10.1038/nrclinonc.2016.66 PMC5461122

[13] Gong Y, Ji P, Yang YS, Xie S, Yu TJ, Xiao Y, Jin ML, Ma D, Guo LW, Pei YC, et al. Therapeutic Targets Cell Metab. 2021;33(1):51–64. 10.1016/j.cmet.2020.10.012. Metabolic-Pathway-Based Subtyping of Triple-Negative Breast Cancer Reveals Potential. 33181091 10.1016/j.cmet.2020.10.012

[14] Brown RE, Short SP, Williams CS. Colorectal cancer and metabolism. Curr Colorectal Cancer Rep. 2018;14(6):226–41. 10.1007/s11888-018-0420-y. 31406492 10.1007/s11888-018-0420-y PMC6690608

[15] La Vecchia S, Sebastián C. Metabolic pathways regulating colorectal cancer initiation and progression. Semin Cell Dev Biol. 2020;98:63–70. 10.1016/j.semcdb.2019.05.018. 31129171 10.1016/j.semcdb.2019.05.018

[16] Martinez-Outschoorn UE, Peiris-Pagés M, Pestell RG, Sotgia F, Lisanti MP. Cancer metabolism: a therapeutic perspective. Nat Rev Clin Oncol. 2017;14(2):113. 10.1038/nrclinonc.2017.1. 27141887 10.1038/nrclinonc.2016.60

[17] Jézéquel P, Loussouarn D, Guérin-Charbonnel C, Campion L, Vanier A, Gouraud W, Lasla H, Guette C, Valo I, Verrièle V, et al. Gene-expression molecular subtyping of triple-negative breast cancer tumours: importance of immune response. Breast Cancer Res. 2015;17:43. 10.1186/s13058-015-0550-y. 25887482 10.1186/s13058-015-0550-y PMC4389408

[18] Sabatier R, Finetti P, Adelaide J, Guille A, Borg JP, Chaffanet M, Lane L, Birnbaum D, Bertucci F. Down-regulation of ECRG4, a candidate tumor suppressor gene, in human breast cancer. PLoS ONE. 2011;6(11):e27656. 10.1371/journal.pone.0027656. 22110708 10.1371/journal.pone.0027656 PMC3218004

[19] Sabatier R, Finetti P, Cervera N, Lambaudie E, Esterni B, Mamessier E, Tallet A, Chabannon C, Extra JM, Jacquemier J, et al. A gene expression signature identifies two prognostic subgroups of basal breast cancer. Breast Cancer Res Treat. 2011;126(2):407–20. 10.1007/s10549-010-0897-9. 20490655 10.1007/s10549-010-0897-9

[20] Ogata H, Goto S, Sato K, Fujibuchi W, Bono H, Kanehisa M. KEGG: Kyoto encyclopedia of genes and genomes. Nucleic Acids Res. 1999;27(1):29–34. 10.1093/nar/27.1.29. 9847135 10.1093/nar/27.1.29 PMC148090

[21] Anders S, Huber W. Differential expression analysis for sequence count data. Genome Biol. 2010;11(10):R106. 10.1186/gb-2010-11-10-r106. 20979621 10.1186/gb-2010-11-10-r106 PMC3218662

[22] Yu G, Wang LG, Han Y, He QY. ClusterProfiler: an R package for comparing biological themes among gene clusters. Omics. 2012;16(5):284–7. 10.1089/omi.2011.0118. 22455463 10.1089/omi.2011.0118 PMC3339379

[23] Subramanian A, Tamayo P, Mootha VK, Mukherjee S, Ebert BL, Gillette MA, Paulovich A, Pomeroy SL, Golub TR, Lander ES, et al. Gene set enrichment analysis: a knowledge-based approach for interpreting genome-wide expression profiles. Proc Natl Acad Sci U S A. 2005;102(43):15545–50. 10.1073/pnas.0506580102. 16199517 10.1073/pnas.0506580102 PMC1239896

[24] Friedman J, Hastie T, Tibshirani R. Regularization paths for generalized linear models via coordinate descent. J Stat Softw. 2010;33(1):1–22. 20808728 PMC2929880

[25] Tian W, Luo Y, Tang Y, Kong Y, Wu L, Zheng S, Zou Y, Zhang C, Xie J, Deng X, et al. Novel implication of the basement membrane for breast cancer outcome and immune infiltration. Int J Biol Sci. 2023;19(5):1645–63. 10.7150/ijbs.81939. 37056938 10.7150/ijbs.81939 PMC10086744

[26] Zou Y, Xie J, Zheng S, Liu W, Tang Y, Tian W, Deng X, Wu L, Zhang Y, Wong CW, et al. Leveraging diverse cell-death patterns to predict the prognosis and drug sensitivity of triple-negative breast cancer patients after surgery. Int J Surg. 2022;107:106936. 10.1016/j.ijsu.2022.106936. 36341760 10.1016/j.ijsu.2022.106936

[27] Therneau TM, Grambsch PM. Modeling Survival Data: Extending the Cox Model. In: 2000; 2000.

[28] Mayakonda A, Lin DC, Assenov Y, Plass C, Koeffler HP. Maftools: efficient and comprehensive analysis of somatic variants in cancer. Genome Res. 2018;28(11):1747–56. 10.1101/gr.239244.118. 30341162 10.1101/gr.239244.118 PMC6211645

[29] Maeser D, Gruener RF, Huang RS. OncoPredict: an R package for predicting in vivo or cancer patient drug response and biomarkers from cell line screening data. Brief Bioinform. 2021;22(6). 10.1093/bib/bbab260. 10.1093/bib/bbab260 PMC8574972 34260682

[30] Newman AM, Steen CB, Liu CL, Gentles AJ, Chaudhuri AA, Scherer F, Khodadoust MS, Esfahani MS, Luca BA, Steiner D, et al. Determining cell type abundance and expression from bulk tissues with digital cytometry. Nat Biotechnol. 2019;37(7):773–82. 10.1038/s41587-019-0114-2. 31061481 10.1038/s41587-019-0114-2 PMC6610714

[31] Tekpli X, Lien T, Røssevold AH, Nebdal D, Borgen E, Ohnstad HO, Kyte JA, Vallon-Christersson J, Fongaard M, Due EU, et al. An independent poor-prognosis subtype of breast cancer defined by a distinct tumor immune microenvironment. Nat Commun. 2019;10(1):5499. 10.1038/s41467-019-13329-5. 31796750 10.1038/s41467-019-13329-5 PMC6890706

[32] Liu P, Wang Z, Ou X, Wu P, Zhang Y, Wu S, Xiao X, Li Y, Ye F, Tang H. The FUS/circEZH2/KLF5/ feedback loop contributes to CXCR4-induced liver metastasis of breast cancer by enhancing epithelial-mesenchymal transition. Mol Cancer. 2022;21(1):198. 10.1186/s12943-022-01653-2. 36224562 10.1186/s12943-022-01653-2 PMC9555172

[33] Tang H, Huang X, Wang J, Yang L, Kong Y, Gao G, Zhang L, Chen ZS, Xie X. circKIF4A acts as a prognostic factor and mediator to regulate the progression of triple-negative breast cancer. Mol Cancer. 2019;18(1):23. 10.1186/s12943-019-0946-x. 30744636 10.1186/s12943-019-0946-x PMC6369546

[34] Gilep AA, Sushko TA, Usanov SA. At the crossroads of steroid hormone biosynthesis: the role, substrate specificity and evolutionary development of CYP17. Biochim Biophys Acta. 2011;1814(1):200–9. 10.1016/j.bbapap.2010.06.021. 20619364 10.1016/j.bbapap.2010.06.021

[35] Favennec L, Cals MJ. The biological effects of retinoids on cell differentiation and proliferation. J Clin Chem Clin Biochem. 1988;26(8):479–89. 10.1515/cclm.1988.26.8.479. 3065440 10.1515/cclm.1988.26.8.479

[36] Peng L, Zhou L, Li H, Zhang X, Li S, Wang K, Yang M, Ma X, Zhang D, Xiang S, et al. Hippo-signaling-controlled MHC class I antigen processing and presentation pathway potentiates antitumor immunity. Cell Rep. 2024;43(4):114003. 10.1016/j.celrep.2024.114003. 38527062 10.1016/j.celrep.2024.114003

[37] Sivori S, Pende D, Quatrini L, Pietra G, Della Chiesa M, Vacca P, Tumino N, Moretta F, Mingari MC, Locatelli F, et al. NK cells and ILCs in tumor immunotherapy. Mol Aspects Med. 2021;80:100870. 10.1016/j.mam.2020.100870. 32800530 10.1016/j.mam.2020.100870

[38] Li Y, Zhang H, Merkher Y, Chen L, Liu N, Leonov S, Chen Y. Recent advances in therapeutic strategies for triple-negative breast cancer. J Hematol Oncol. 2022;15(1):121. 10.1186/s13045-022-01341-0. 36038913 10.1186/s13045-022-01341-0 PMC9422136

[39] Liu Y, Su Z, Tavana O, Gu W. Understanding the complexity of p53 in a new era of tumor suppression. Cancer Cell. 2024;42(6):946–67. 10.1016/j.ccell.2024.04.009. 38729160 10.1016/j.ccell.2024.04.009 PMC11190820

[40] Ghelli Luserna di Rorà, Cerchione A, Martinelli C, Simonetti G. A WEE1 family business: regulation of mitosis, cancer progression, and therapeutic target. J Hematol Oncol. 2020;13(1):126. 10.1186/s13045-020-00959-2. 32958072 10.1186/s13045-020-00959-2 PMC7507691

[41] Platten M, Nollen EAA, Röhrig UF, Fallarino F, Opitz CA. Tryptophan metabolism as a common therapeutic target in cancer, neurodegeneration and beyond. Nat Rev Drug Discov. 2019;18(5):379–401. 10.1038/s41573-019-0016-5. 30760888 10.1038/s41573-019-0016-5

[42] Sun C, Li M, Zhang L, Sun F, Chen H, Xu Y, Lan Y, Zhang L, Lu S, Zhu J, et al. IDO1 plays a tumor-promoting role via MDM2-mediated suppression of the p53 pathway in diffuse large B-cell lymphoma. Cell Death Dis. 2022;13(6):572. 10.1038/s41419-022-05021-2. 35760783 10.1038/s41419-022-05021-2 PMC9237101

[43] Gargaro M, Manni G, Scalisi G, Puccetti P, Fallarino F. Tryptophan metabolites at the crossroad of Immune-Cell interaction via the Aryl hydrocarbon receptor: implications for tumor immunotherapy. Int J Mol Sci. 2021;22(9). 10.3390/ijms22094644. 10.3390/ijms22094644 PMC8125364 33924971

