# Supplementary material for: Comprehensive analysis of metabolism-related gene biomarkers reveals their impact on the diagnosis and prognosis of triple-negative breast cancer
Source: BMC Cancer. 2025 Apr 11;25:668. doi: 10.1186/s12885-025-14053-8 (PMC11987350; doi:10.1186/s12885-025-14053-8)
Supplement: Supplementary file 5 — Supplementary Material 5 [file 12885_2025_14053_MOESM5_ESM.docx]

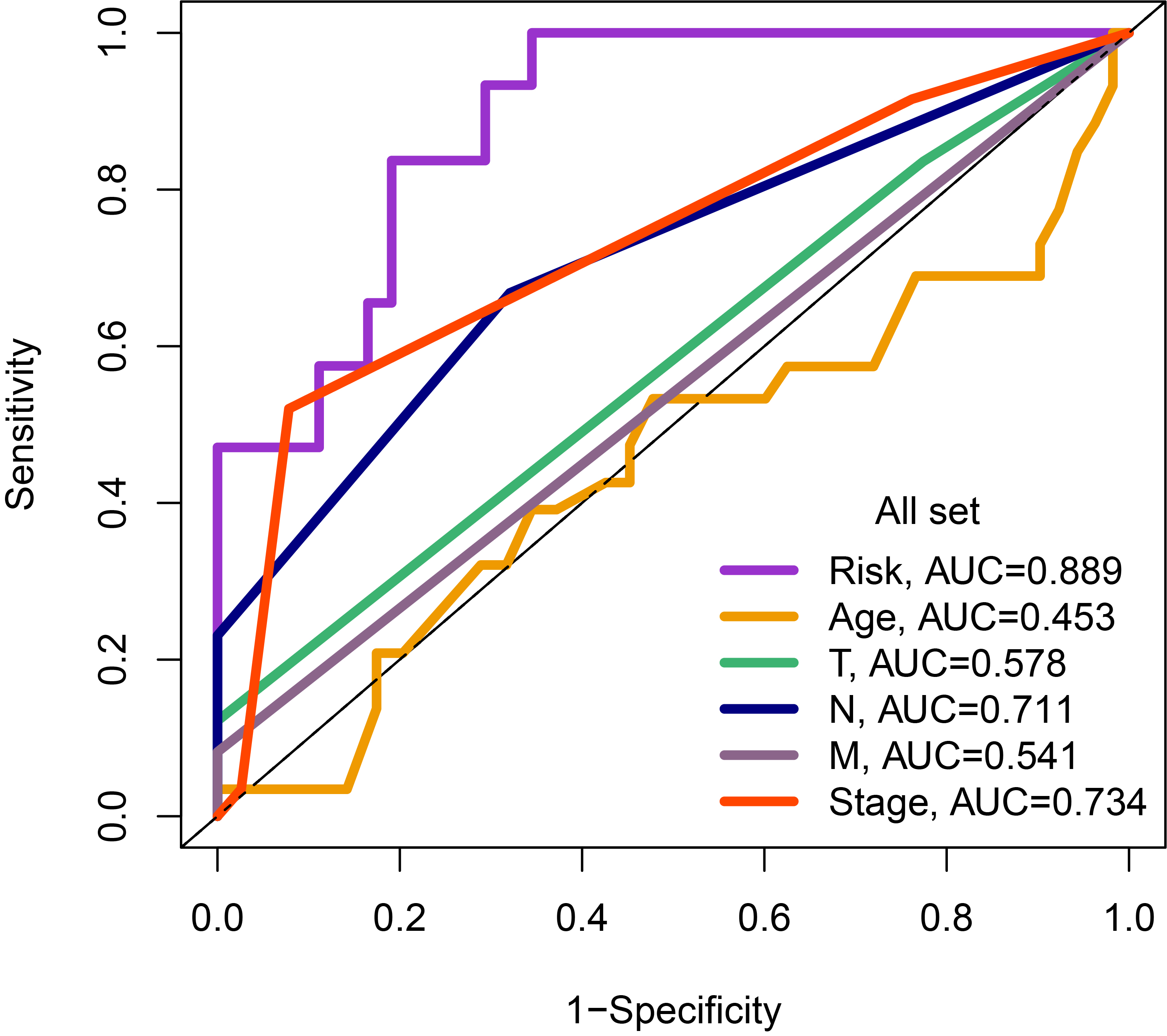


Supplementary Figures 1. ROC Curves Demonstrating the Predictive Efficacy of Metabolism-Related Gene Risk Score and Its Combination with Clinical Parameters.


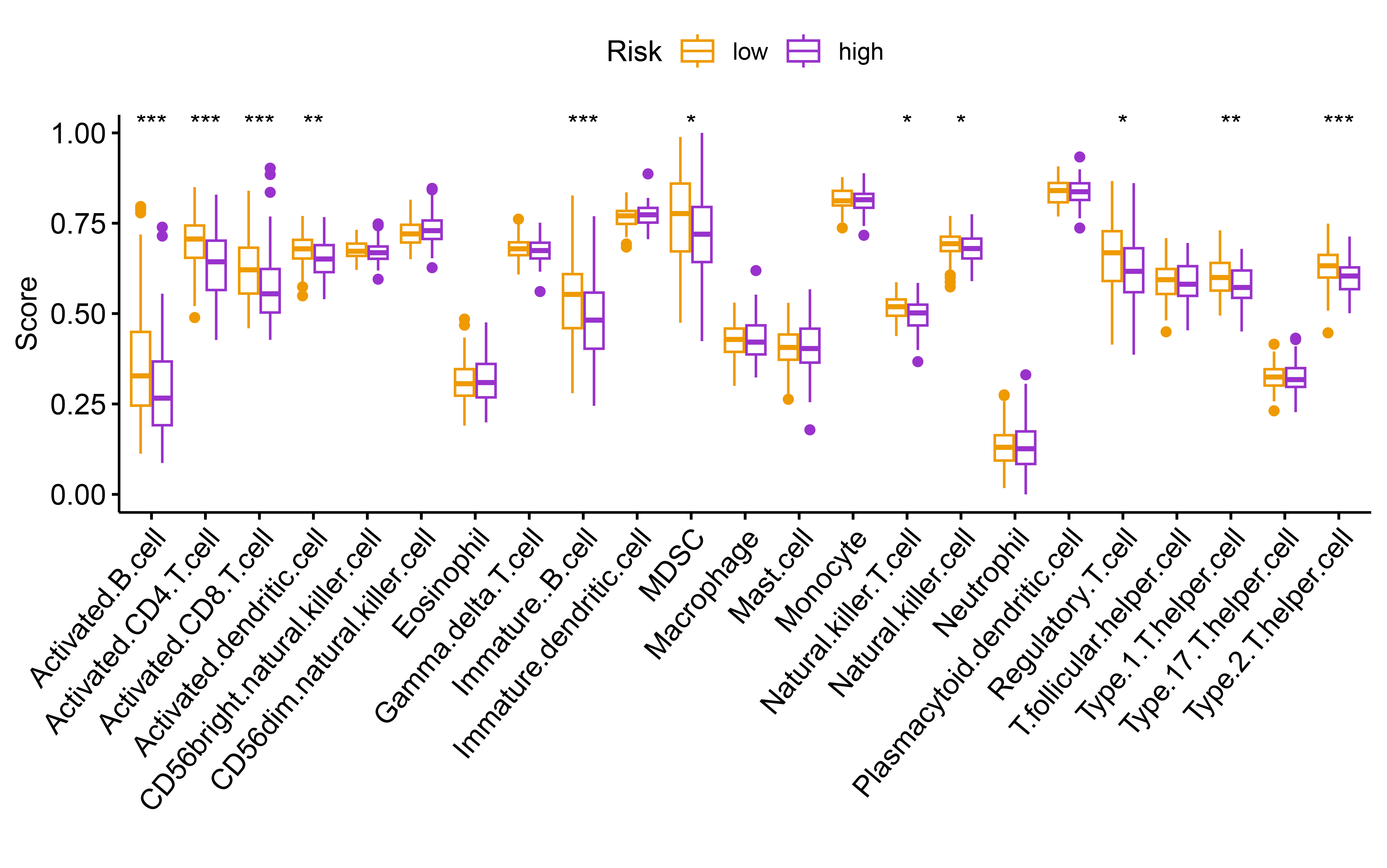


Supplementary Figures 2. Comparison of Immune Cell Activity Scores Between High and Low Risk Groups by ssGSEA. Statistical significance is marked as: p < 0.05 (*), p < 0.01 (**), p <0.001 (***), p < 0.0001 (****).


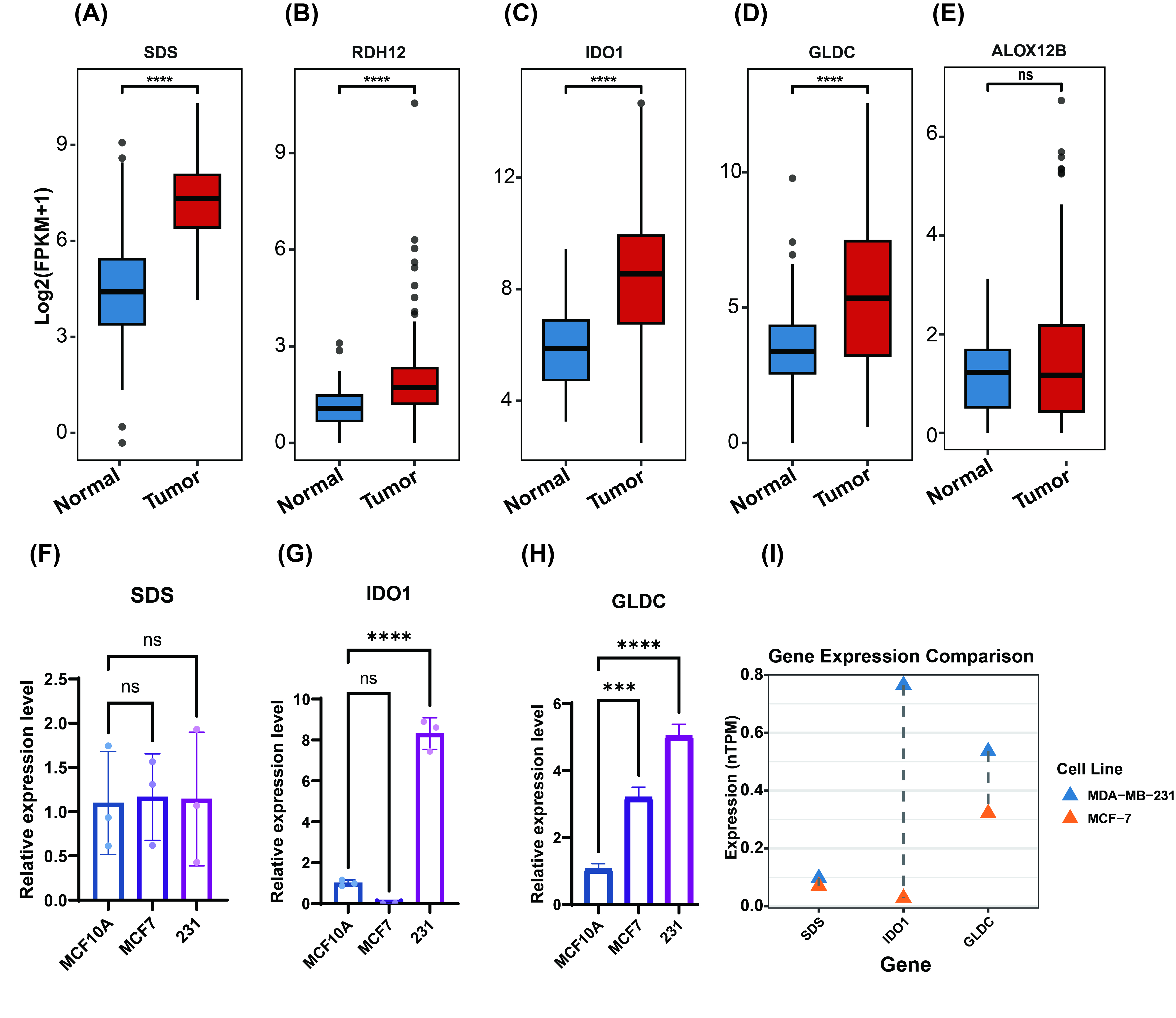


Supplementary Figure 3. Analysis Expression of key genes in the breast cancer prognostic model (A)-(E): Comparison of mRNA expression levels of SDS, RDH12, IDO1, GLDC, and ALOX12B genes between triple-negative breast cancer (TNBC) tissues and normal breast tissues. Data are based on RNA-Seq (Log2(FPKM+1)). Statistical significance is marked as: p < 0.05 (*), p < 0.01 (**), p <0.001 (***), p < 0.0001 (****). (F)-(H): Relative expression levels of SDS, IDO1, and GLDC genes in representative breast cancer cell lines (MDA-MB-231, MCF-7) and normal breast epithelial cells (MCF-10A). Statistical significance is marked as: p < 0.05 (*), p < 0.01 (**), p <0.001 (***), p < 0.0001 (****). (I): Analysis of SDS, IDO1, and GLDC gene expression levels based on the CCLE (Cancer Cell Line Encyclopedia) database.


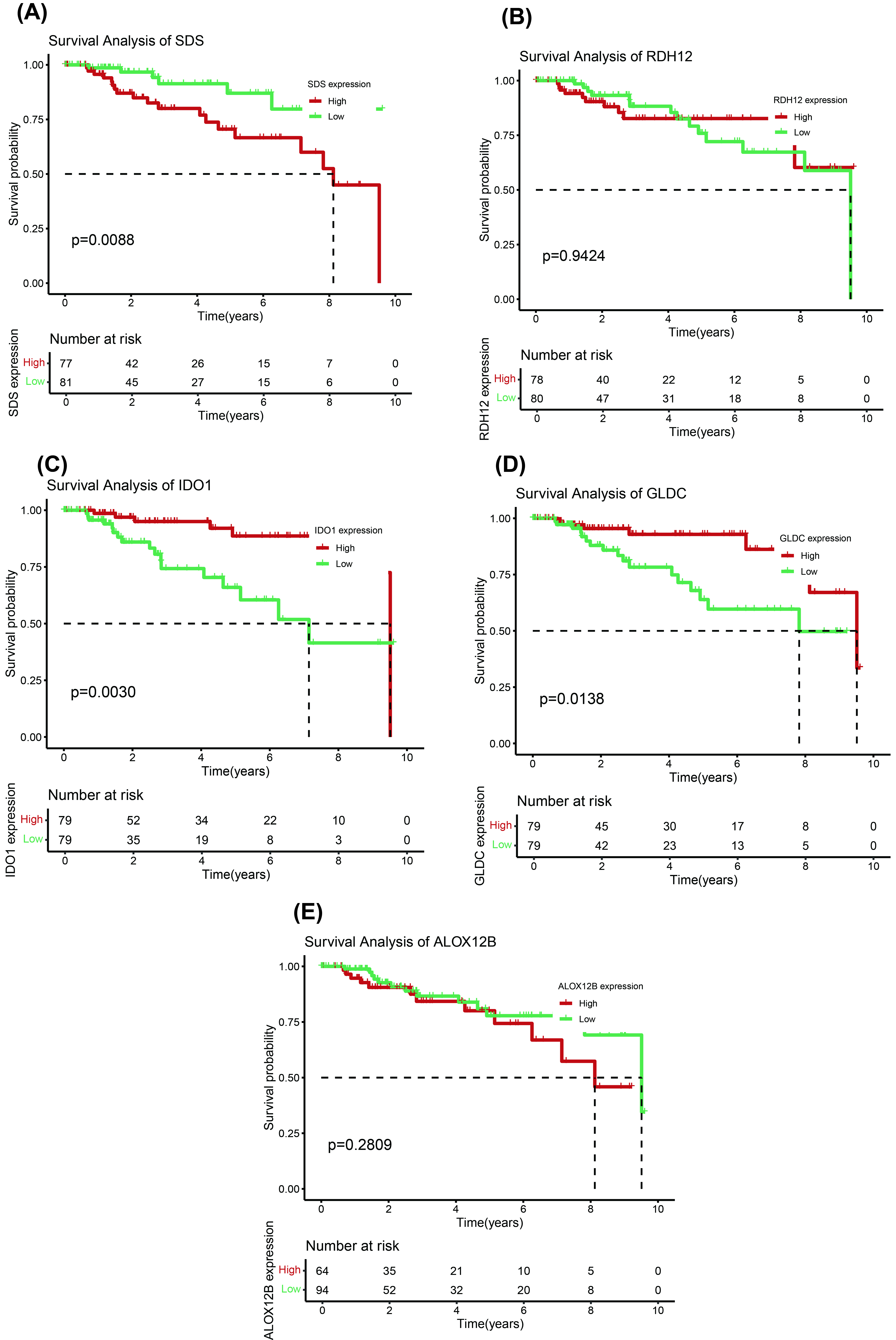


Supplementary Figure 4. Survival Analysis of Five Key Genes in TCGA-TNBC Cohort. All panels (A-E) present Kaplan-Meier survival analyses for SDS, RDH12, IDO1, GLDC, and ALOX12B in the TCGA-TNBC cohort. In each panel, the red line represents the high expression group and the green line represents the low expression group. The p-value for each analysis is indicated within the respective panel.
